# Crystal Structures of Lysine-Preferred Racemases, the Non-Antibiotic Selectable Markers for Transgenic Plants

**DOI:** 10.1371/journal.pone.0048301

**Published:** 2012-10-31

**Authors:** Hsin-Mao Wu, Yi-Chia Kuan, Chia-Han Chu, Wen-Hwei Hsu, Wen-Ching Wang

**Affiliations:** 1 Institute of Molecular and Cellular Biology and Department of Life Science, National Tsing Hua University, Hsinchu, Taiwan; 2 Institute of Molecular Biology, National Chung Hsing University, Taichung, Taiwan; 3 Biomedical Science and Engineering Center, National Tsing Hua University, Hsinchu, Taiwan; Concordia University Wisconsin, United States of America

## Abstract

Lysine racemase, a pyridoxal 5′-phosphate (PLP)-dependent amino acid racemase that catalyzes the interconversion of lysine enantiomers, is valuable to serve as a novel non-antibiotic selectable marker in the generation of transgenic plants. Here, we have determined the first crystal structure of a lysine racemase (Lyr) from *Proteus mirabilis* BCRC10725, which shows the highest activity toward lysine and weaker activity towards arginine. In addition, we establish the first broad-specificity amino acid racemase (Bar) structure from *Pseudomonas putida* DSM84, which presents not only the highest activity toward lysine but also remarkably broad substrate specificity. A complex structure of Bar-lysine is also established here. These structures demonstrate the similar fold of alanine racemase, which is a head-to-tail homodimer with each protomer containing an N-terminal (α/β)_8_ barrel and a C-terminal β-stranded domain. The active-site residues are located at the protomer interface that is a funnel-like cavity with two catalytic bases, one from each protomer, and the PLP binding site is at the bottom of this cavity. Structural comparisons, site-directed mutagenesis, kinetic, and modeling studies identify a conserved arginine and an adjacent conserved asparagine that fix the orientation of the PLP O3 atom in both structures and assist in the enzyme activity. Furthermore, side chains of two residues in α-helix 10 have been discovered to point toward the cavity and define the substrate specificity. Our results provide a structural foundation for the design of racemases with pre-determined substrate specificity and for the development of the non-antibiotic selection system in transgenic plants.

## Introduction

Certain bacteria synthesize D-amino acids, e.g., D-Ala, D-Glu, D-Val, and D-Phe, for metabolic processes [1], [2]. Interconversion of D- and L-amino acid enantiomers occurs not only in bacteria [3], [4], [5] but also in eukaryotes [6], [7].

Two types of amino acid racemases are known: pyridoxal 5′-phosphate (PLP)-dependent [6] and PLP-independent enzymes [8]. Alanine racemase (Alr, E.C. 5.1.1.1), which catalyzes the interconversion of D- and L-Ala, is the best characterized of the prokaryotic and eukaryotic PLP-dependent enzymes. Several Alr crystal structures have been determined, and the structures are compact homodimers with each protomer containing an (α/β)_8_ barrel and a C-terminal domain having three β-sheets [9], [10], [11], [12], [13], [14]. The dimeric interface contains the active-site cleft, which is formed by residues from the (α/β)_8_ barrel of one protomer and residues from the C-terminal domain of the other protomer. Structural and site-directed mutagenesis studies of *Bacillus stearothermophilus* Alr (BsAlr, PDB code: 1L6G) have identified two catalytic residues: Lys^39^ from one subunit and Tyr^265^ from the other subunit [13], [15], [16], [17]. When alanine is absent, the C4A atom of PLP forms an aldimine bond with the Lys39 Nζ of BsAlr, whereas PLP forms an aldimine bond with the amino nitrogen of the alanine substrate during catalysis [9], [13].

A constricted entryway has been characterized in *Mycobacterium tuberculosis* Alr (MtAlr; PDB code: 1XFC) [18] that is believed to be the active site. The entryway is formed by three layers of residues (outer layer: Asp^357^, Lys^178^, and Ala^241^; middle layer: Arg^316′^, Ile^362^, Arg^296′^, and Asp^177^; inner layer: Tyr^271′^, Tyr^364^, Tyr^290′^, and Ala^176^) (prime symbol represents the residue from the other subunit of a dimer). After the entryway, PLP is found at the base of the cavity, and importantly, the strict Alr specificity for alanine is defined by the relatively small separation (∼2.7 Å) between two innermost layer tyrosines: MtAlr, Tyr^271′^ and Tyr^364^; *Streptococcus pneumoniae* Alr (SpAlr, PDB code: 3S46 [19]), Tyr^263′^ and Tyr^352^; BsAlr, Tyr^265′^and Tyr^354^ [18].

In addition to Alrs, a PLP-dependent racemase is found to possess a broad amino-acid specificity (broad specificity amino acid racemase, Bar, E.C. 5.1.1.10 [20], [21]). Moreover, several microorganisms, including *Proteus vulgaris* [22], *P. putida* [23], *Proteus spp.* and *Escherichia spp.* [24], have lysine racemization activities, although the genes encoding lysine racemases and the lysine racemases themselves have not been isolated. Recently, *lyr* encoding a lysine racemase was obtained from a library constructed from the genetic material of garden soil microbes [25]. By screening a *P. mirabilis* BCRC10725 genomic library, a *lyr* was found that complemented a lysine-auxotrophic *P. mirabilis* mutant cultured on M9-containing agar supplemented with D-lysine [26].

Of note, the sequences of Bar and Lyr contain conserved lysine and tyrosine residues at positions equivalent to the catalytic residues of BsAlr (Bar: Lys^75^, Tyr^301′^; Lyr: Lys^74^, Tyr^299′^), despite their otherwise limited sequence identity (28% between Bar and BsAlr; 24% between Lyr and BsAlr). Furthermore, the *P. mirabilis* BCRC10725 *lyr* sequence is identical with *P. mirabilis* ATCC29906 gene (NCBI accession number: ZP 03840526), which is annotated as a putative alanine racemase. According to a phylogenetic study that used Molecular Evolutionary Genetics Analysis, 24 prokaryotic amino acid racemases from 18 species have been classified into different groups: Group I contains Alrs from Gram-negative bacteria, Group II contains Alrs from Gram-positive bacteria, and Group III are non-Alr racemases, which have evolutionarily diverged from group I and II Alrs [26]. Lyr and Bar are therefore Group-III racemases. Expression and biochemical characterization of recombinant Lyr has shown that its preferred substrate is lysine [26]. Notably, Lyr is also active against arginine, whereas Bar racemizes arginine, lysine, methionine, serine, cysteine, leucine, and histidine [27], a useful feature for biotechnological applications.

It is interesting that *lyr* has been successfully used as a non-antibiotic selectable marker gene for plant transformation lately [28] since L-lysine is toxic to tobacco and Arabidopsis while D-lysine supports their growth [28]. Such a non-antibiotic selection system for transgenic plants is much desirable due to the ecological concerns and food safety issues in this modern world. To further develop *lyr*- or *bar*-based marker genes with desired properties, we determined the three-dimensional structures of *P. putida* DSM84 Bar and *P. mirabilis* Lyr, which are the first structures for lysine-preferred racemases. In addition, the first liganded Bar-lysine structure was also established. All structures contain PLP, which confirms their PLP-dependent nature, and the positions of their catalytic lysines and tyrosines are similar to those in Alr structures. Site-directed mutagenic, kinetic, and modeling studies characterized the Lyr/Bar PLP-binding residues and the residues responsible for their substrate specificities.

## Results and Discussion

### Bar and Lyr Structures

**Figure 1:**
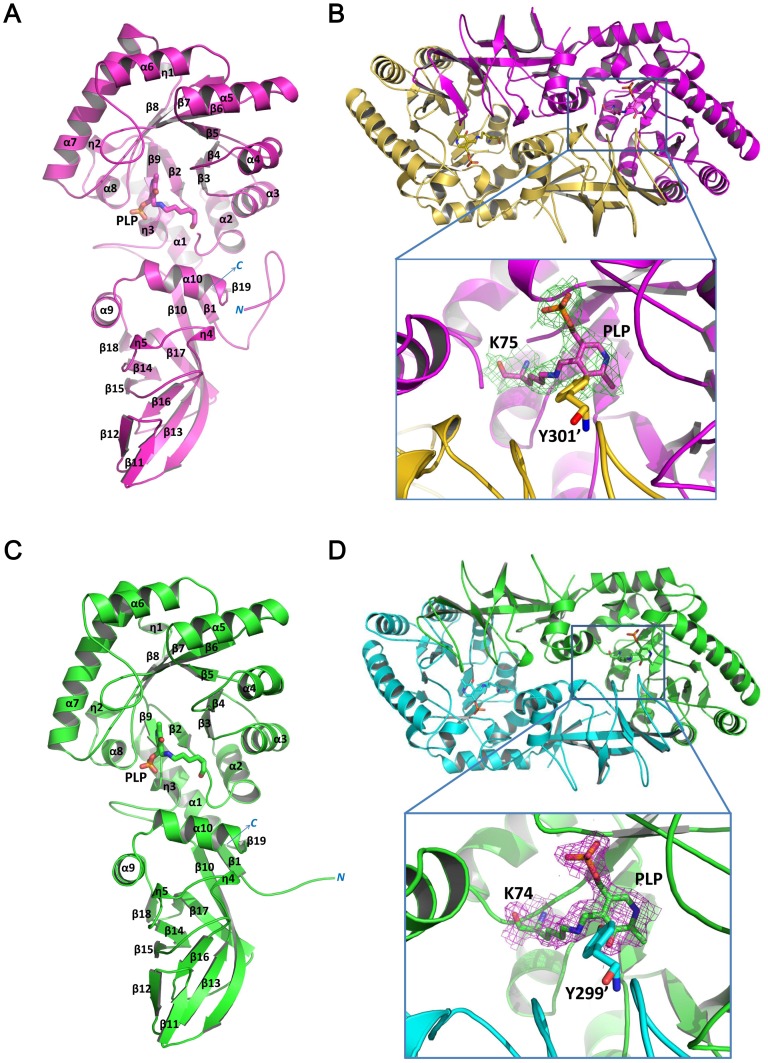
Bar and Lyr Structures. (A) Monomeric Bar. The N- and C-termini are labeled *N* and *C*, respectively, in blue. The α-helices and β-strands are labeled α and β, respectively, and are numbered. The internal aldimine linkage between PLP and K75 is shown as stick. (B) Dimeric Bar. The electron density map for the aldimine bond between PLP and K75, and the other catalyst Y301′ (prime denotes the residue from the other subunit) are shown in the lower panel. (C) Monomeric Lyr. The N- and C-termini are labeled *N* or *C*, respectively, in blue. The α-helices and β-strands are labeled α and β, respectively, and are numbered. The internal aldimine linkage between PLP and K74 is shown as stick. (D) Dimeric Lyr. The electron density map for the aldimine bond between PLP and K74, and the other catalyst Y299′ are shown in the lower panel. The omit *Fo–Fc* electron density maps are contoured at 1.0 σ. Oxygen, nitrogen, and phosphate atoms are colored red, blue, and orange, respectively.

The final Lyr and Bar structures had *R* values of 22.0% (*R_free_* = 26.0%) and 15.8% (*R_free_* = 21.5%), respectively. Refinement statistics are given in Table 1. The asymmetric unit in the Bar crystal contains two molecules. With the exception of the positions for the first 25 residues, those of all other main-chain and side-chain atoms are well defined. Non-peptide electron density near Lys75 was fit with an aldimine bond between the Lys75 Nζ and the PLP C4A. The average B-factor is 37.4 Å^2^. The Bar molecule contains two domains: an N-terminal (α/β)_8_ barrel, which contains PLP, and a β-stranded C-terminal domain (Figure 1A). Two Bar molecules associate to form an AB-type dimer, and their positions are related by a non-crystallographic two-fold axis (Figure 1B). The subunits have essentially the same fold (root mean square deviation (RMSD) = 0.219 Å for equivalent Cα positions).

**Table 1:** X-ray Data and Refinement Statistics.

Data collection and phasing								
Dataset	Lyr	Bar	Complex-Bar	SeMet-Bar (MAD)				
				Peak		Edge	High remote	
Beamline	SPring-8_SP44XU	NSRRC_BL13B1	SPring-8_SP12B2	NSRRC_BL13B1				
Space group	C222_1_	C2	C2			C2		
Cell dimensions								
*a* (Å)	62.85	142.25	142.42		140.70	140.90	140.89	
*b* (Å)	85.07	118.74	118.11		118.69	118.94	118.94	
*c* (Å)	151.29	77.78	74.02		73.94	74.16	74.17	
Wavelength (Å)	1.0000	1.0000	1.0000		0.9791	0.9792	0.9638	
Resolution (Å)	30.00−1.74	30.00−2.45	20.00−3.10		30.00−2.10	30.00−2.20	30.00−2.20	
Highest resolution shell (Å)	1.80−1.74	2.54−2.45	3.21−3.10		2.18−2.10	2.28−2.20	2.28−2.20	
Completeness (%)^a^	95.4 (100.0)	99.7 (98.5)	99.8(98.4)		98.8 (93.4)	97.9 (91.7)	98.0 (93.0)	
Average *I/σ (I)* a	21.4 (3.5)	15.4 (4.0)	9.8(3.3)		16.8 (6.4)	13.1 (3.7)	13.5 (3.6)	
No. of unique reflections	39,932	46,692	21,648		69,330	59,991	60,170	
Redundancy^a^	5.1 (5.6)	4.1 (4.0)	4.2(4.1)		7.3 (6.6)	3.6 (3.5)	3.6 (3.5)	
*R_merge_* (%)a, b	6.2 (48.0)	8.9 (46.1)	15.3(40.7)		9.5 (24.6)	8.7 (39.1)	8.8 (41.6)	
Overall figure of merit^c^						0.87		
Solvent content (%)	46.6	65.9	63.9					
**Refinement**								
Resolution range (Å)	30.00−1.74	30.00−2.45	20.00−3.10					
Number of atoms								
Protein	2890	5870	5890					
Solvent	224	381	95					
Average *B*-factor (Å^2^)	29.2	37.4	43.2					
*R* factor^d^	0.220	0.158	0.175					
*R_free_* e	0.260	0.215	0.219					
RMSD bond lengths (Å)^f^	0.020	0.017	0.017					
RMSD bond angles (°)^f^	1.477	1.469	1.376					
Estimated coordinate error (Å)	0.092	0.268	0.271					
Ramachandran analysis (%)^g^FavoredAllowedGenerousDisallowed	88.610.50.90.0	88.111.60.30.0	83.315.80.90.0					

a Values in parentheses refer to statistics in the highest-resolution shell.

b *R_merge_* = Σ|*I*_obs_−<I>|/Σ*I*_obs_.

c Figure of merit = |*F*_best_|/|*F*|.

d *R* = Σ|*F*_obs_-*F*_calc_| / Σ*F*_obs,_ where *F*_obs_ and *F*_calc_ are the observed and calculated structure-factor amplitudes, respectively.

e *R_free_* was computed using 5% of the data assigned randomly.

f Root mean square deviation.

g Estimated standard uncertainties based on maximum likelihood.

We next solved the Lyr structure by molecular replacement with the Bar structure as the template. One Lyr molecule is found in the asymmetric unit. With the exception of those for the first 36 residues, the positions of all main-chain and side-chain atoms are well defined. The average B-factor is 29.2 Å^2^. Molecular Lyr and Bar have the same domain structure, and PLP is found in an equivalent position in the two molecules (Figure 1C). Two Lyr molecules associate to form a dimer related by a two-fold crystallographic axis. In the Bar and Lyr dimers, the two protomers are arranged in a head-to-tail manner, stabilized by multiple interactions between N-terminal-domain residues in one monomer and C-terminal-domain residues in the other protomer (Figure 1B, D). The tertiary structures of the Lyr and Bar protomers are similar (RMSD = 0.89 Å for Cα positions).

### Structural Comparisons

When the Lyr or Bar structure was used as the query, the top-scoring proteins (Z scores >38) returned by DALI were Alrs, even though the sequence identities for the Alrs and Lyr, and the Alrs and Bar are low (20−27% identity for Lyr; 23−31% identity for Bar). The tertiary folds of Lyr, Bar, and the Alrs are similar [29], and the protomers in each racemase are oriented head-to-tail.

**Figure 2:**
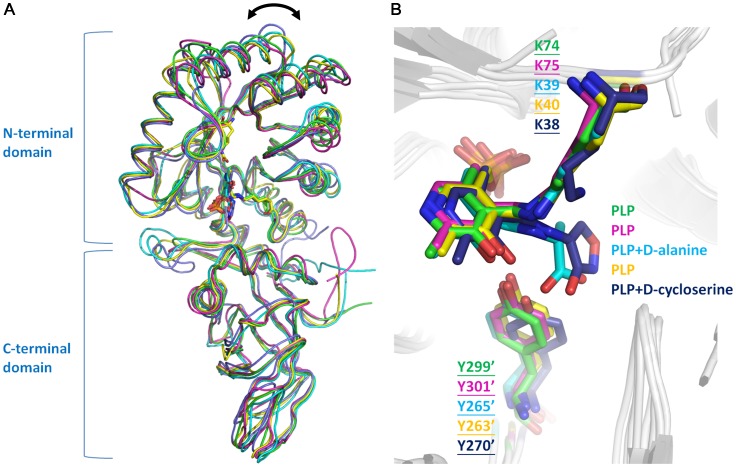
Superposition of Lyr, Bar, BsAlr, SpAlr and SlAlr. Lyr, Bar, BsAlr (PDB code: 1L6G), SpAlr (PDB code: 3S46), and SlAlr (PDB code: 1VFS) are colored green, magenta, cyan, yellow, and slate, respectively. (A) Superpositioning of the protomers. The variation of hinge angles between the monomer domains is indicated by the double-headed arrow. (B) The two conserved catalytic residues, PLP, and substrates are shown as stick models. Oxygen, nitrogen, and phosphate atoms are colored red, blue, and orange, respectively.

**Figure 3:**
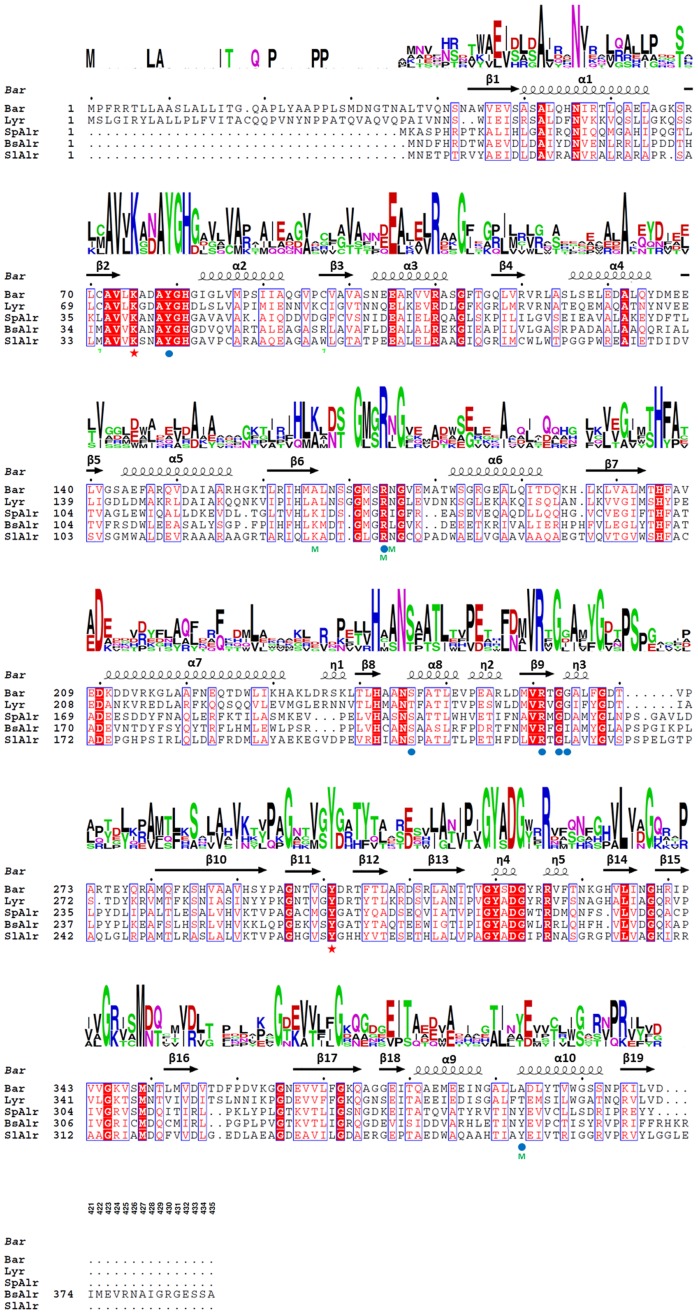
Sequence Alignment of Bar, Lyr, SpAlr, BsAlr, and SlAlr. The catalytic residues, PLP-binding residues, and the mutation sites are identified with a red star, blue circle, and m (green), respectively. The relative conservation of each residue was drawn by the web-server WEBLOGO (http://weblogo.berkeley.edu/).

Superposition of Lyr, Bar, SpAlr, BsAlr, and SlAlr confirmed their structural similarity (Figure 2A). The C-terminal domains superimpose relatively well (Q-scores within 0.68–0.95 for Cα positions) [30]. However, the Cα positions of a few regions in the N-terminal domains (corresponding to residues 108−121, 149−155, and 191−195 in Lyr) have lower structural similarities between our structures and Alrs (Q-scores within 0.45–0.60). Moreover, the Cα positions in the η3-β10 loop between the N- and C-terminal domains have the greatest deviation (Q-scores within 0.38–0.45) and show that the hinge angles between the monomer domains are slightly different (Figure 2A), which are also discovered in Alr structures [14], [19]. Lyr Lys^74^ and Bar Lys^75^ reside in the β2-α2 loop and bind PLP via an aldimine bond as has been found for the corresponding lysines and PLP in the Alrs (Figure 2B). The combined sequence and secondary structure alignments of Lyr, Bar, and the three Alrs demonstrate that this loop along with β2, α2, β11, β11–β12, and β12 share similar secondary structural elements (Figure 3). Additionally, the Cα atoms in the sequences of these regions in Lyr and Bar superimpose very well with those in the Alrs (Q-scores within 0.85–0.95). Therefore, Lyr, Bar, and the Alrs probably originated from a common ancestor involved in racemization of amino acids for the synthesis of cell building blocks.

### Racemase Active Sites

The Alr active-site cleft is located at the dimeric interface and contains the two conserved catalytic residues, a lysine from one subunit and a tyrosine from the other subunit (SpAlr: Lys^40^ and Tyr^263′^; BsAlr: Lys^39^ and Tyr^265′^; SlAlr: Lys^38^ and Tyr^270′^ [9], [11], [31]). Sequence alignment of Lyr, Bar, and the Alrs showed that the two catalytic residues are conserved in Lyr and Bar (Figure 2B). Furthermore, superpositioning the Lyr, Bar, SpAlr, BsAlr, and SlAlr structures showed that the two catalytic residues are situated at nearly identical positions within all five structures (Figure 1, 2, 3).

**Figure 4:**
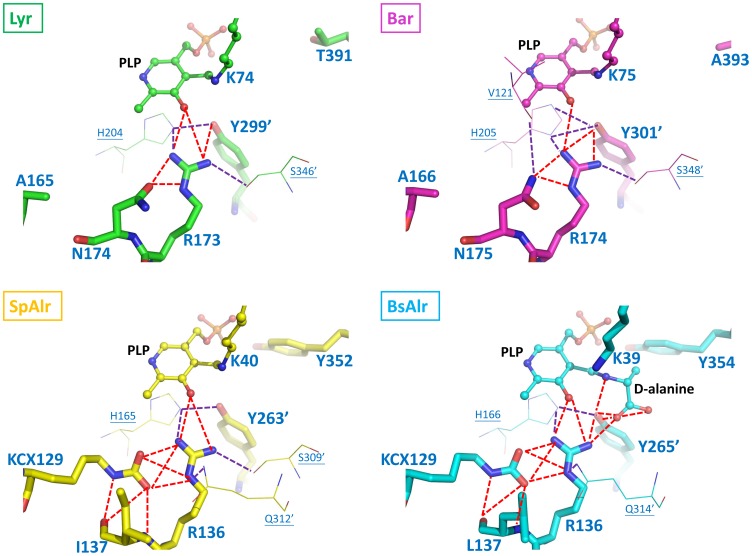
The Hydrogen-bonding Networks in the Binding Pockets of Lyr, Bar, SpAlr, and BsAlr. The carbon atoms of Lyr, Bar, SpAlr, and BsAlr are shown in green, magenta, yellow, and cyan, respectively. The views are those obtained by superpositioning the structures. The aldimine linkages involving PLP and K74 in Lyr, PLP and K75 in Bar, PLP and K40 in SpAlr, and PLP and d-Ala in BsAlr are shown as ball-and-stick models. Protein residues are shown as stick models. Oxygen, nitrogen, and phosphate atoms are colored red, blue, and orange, respectively. The hydrogen bonds between a ligand and the four residues KCX129, R136, L137, and Y265′ in BsAlr are shown as dashed red lines, and other hydrogen bonds are shown as dashed purple lines.

Most of the Alr PLP-binding residues are also conserved in Lyr and Bar (Table 2). Two conserved arginines (Lyr: Arg^173^ and Arg^260^; Bar: Arg^174^ and Arg^261^; SpAlr: Arg^136^ and Arg^218^; BsAlr: Arg^136^ and Arg^219^; SlAlr: Arg^136^ and Arg^224^) in the (α/β)_8_ barrel interact with the pyridine N1 and the phenolic O (O3) of PLP, respectively, and they orientate the PLP phenolic ring. A conserved lysine in SpAlr/BsAlr (Lys^129^) can be carbamylated at its Nζ (KCX) and interacts electrostatically with the guanidinium group of Arg^136^, thereby further stabilizing the position of PLP [12], [14]. However, an equivalent lysine is not found in Lyr or Bar (Figure 4). Instead, the side chain of the asparagine that is adjacent to the corresponding arginine (Lyr: Asn^174^; Bar: Asn^175^) makes contacts with the arginine guanidinium moiety in Lyr and Bar, suggesting that these Asn/Arg interactions stabilize the orientation of PLP.

**Table 2:** Contacts between PLP and Its Binding Residues in the Five Racemases.

RacemasesAtoms of PLP	Lyr	Bar	SpAlr	SlAlr	BsAlr
**O1P**	Thr245 N^a^	Ser246 N^a^	Ser203 N^a^	Ser209 N^a^	Ser204 N^a^
	Thr245 OG1^a^	Ser246 OG^a^	Ser203 OG^a^	Ser209 OG^a^	Ser204 OG^a^
	Gly262 N^a^	Gly263 N^a^	Gly220 N^b^	Gly226 N^a^	Gly221 N^a^
		Gly264 N^a^			Ile222 N^b^
**O2P**	Tyr78 OH^a^	Tyr79 OH^a^	Tyr44 OH^a^	Tyr42 OH^a^	Tyr43 OH^a^
	Gly263 N^a^	Gly264 N^a^	Asp221 N^a^	Leu227 N^a^	Ile222 N^a^
**O3P**	(2 H_2_O)	(2 H_2_O)	Tyr352 OH^a^	Tyr361 OH^a^	Tyr354 OH^a^
**O3**	Arg173 NH2^b^	Gol O2^a^	Arg136 NH2^a^	Arg136 NH1^a^	Lys39 NZ^a^
		Arg174 NH2^b^			Arg136 NH2^a^
					Arg136 NH1^b^
**N1**	Arg260 NE^a^	Arg261 NE^a^	Arg218 NE^a^	Arg224 NE^a^	Arg219 NE^a^
	Arg260 NH2^b^	Arg261 NH2^b^	Arg218 NH2^b^	Arg224 NH2^b^	Arg219 NH1^b^

a <3.3 Å.

b 3.3–3.5 Å.

### Lyr and Bar Substrate Binding Cavities

**Figure 5:**
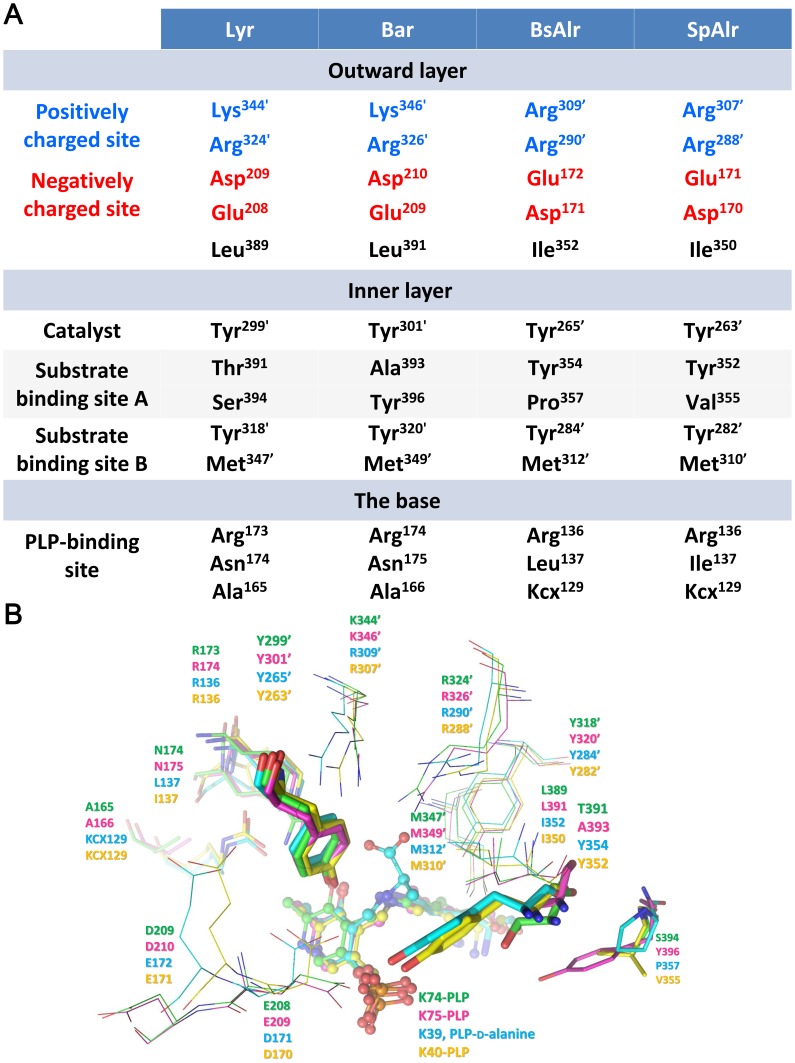
Crucial residues of binding cavities (See also Figure S1). (A) Crucial residues of Lyr, Bar, SpAlr and BsAlr in the binding cavities. (B) Superposition of binding cavities in Lyr (green), Bar (magenta), SpAlr (yellow), and BsAlr (cyan), respectively. Crucial residues are displayed as stick models. The ligands presented in binding pockets of all structures are drawn as ball-and-stick models. Oxygen, nitrogen, and phosphate atoms are colored red, blue, and orange, respectively.

A funnel-like cavity (Figure S1) consisting of inner and outward layers is seen for Lyr or Bar (Figure 5), an arrangement that is also found in MtAlr [18]. An inner-layer tyrosine located in α-helix 10 (Tyr^352^ in SpAlr; Tyr^354^ in BsAlr) interacts with the PLP O3P–an interaction believed to be necessary for substrate specificity [32]. Together with the catalytic tyrosine, these two tyrosine residues (SpAlr: Tyr^352^ and Tyr^263′^; BsAlr: Tyr^354^ and Tyr^265′^), which are separated by ∼2.7 Å, are strictly conserved and orient the alanine substrate and PLP properly. The α-helix 10 Tyr is not, however, present in Lyr and Bar, but is replaced by Thr^391^ in Lyr and Ala^393^ in Bar (Figure 4−5). Two water molecules contact the PLP O3P in Lyr and Bar, filling the space occupied by the tyrosine and PLP in SpAlr and BsAlr (Figure 4). Given the relatively larger space between Thr^391^ and PLP in Lyr, and Ala^393^ and PLP in Bar as compared with those in the Alrs, a substrate larger than an alanine may better fit the space.

For *E. coli* Alr, negatively and positively charged residues are found on the opposite sides of the surface of its cavity surface (Asp^164^, Glu^165^ and Arg^280′^, Arg^300′^) [33]. This array may help properly orientate the substrate for catalysis [33]. Superpositioning the corresponding residues in Lyr (Glu^208^, Asp^209^, Arg^324′^, and Lys^344′^), Bar (Glu^209^, Asp^210^, Arg^326′^, and Lys^346′^), SpAlr (Asp^170^, Glu^171^, Arg^288′^, and Arg^307′^), and BsAlr (Asp^171^, Glu^172^, Arg^290′^, and Arg^309′^) revealed that the charge distribution is similar in the four racemases (Figure 5, see also Figure S1), which supports the suggestion that these residues orient the substrate for catalysis.

### Effects of Mutations on Enzymatic Activity

To evaluate the roles of Arg^173^, Asn^174^, and Thr^391^ in Lyr and Arg^174^, Asn^175^, and Ala^393^ in Bar (corresponding to Arg^136^, Leu^137^, and Tyr^354^ in BsAlr, respectively), we performed site-directed mutagenesis and characterized the effects of the mutations had on racemase activity. The conserved Arg^173^ in Lyr (Arg^174^ in Bar) was replaced with an alanine or a lysine. Asn^174^ in Lyr (Asn^175^ in Bar) was replaced with a leucine to mimic Leu^137^ in BsAlr. Thr^391^ in Lyr (Ala^393^ in Bar) was replaced with a tyrosine to mimic the conserved Alr tyrosines (Figure 4; Tyr^354^ in BsAlr and Tyr^352^ in SpAlr). Each mutant was expressed as a (His)_6_-tagged protein in *E. coli* and purified by Ni-affinity chromatography. CD analysis of each mutant revealed nearly identical profile to that of the wild-type enzyme (Figure S2). The racemase activity of each mutant toward L-Lys, L-Arg, and L-Ala was measured.

R173A and R173K were inactive (Table 3) as were the corresponding Bar mutants R174A and R174K, supporting the importance of a conserved arginine at this position. Possibly, the Lyr Arg^173^ guanidinium group orients the PLP pyridine ring, to position the PLP-Tyr^299′^ interaction for catalysis. The positive electrostatic field of the guanidinium group may also help lower the pKa value of the Tyr^299′^ phenolic hydroxyl (pKa = 8.0–9.0) for effective catalysis.

**Table 3:** Racemization Activities toward L-Lys, L-Arg, or L-Ala by Wild-type Lyr and Bar, and Their Mutants.

Enzymes	l-Lys		l-Arg		l-Ala	
	Specific activity (U/mg)	Ratio^a^	Specific activity (U/mg)	Ratio	Specific activity (U/mg)	Ratio
Wild-type Lyr	2813±97	100%	568±28	20%	ND	
Lyr-R173A	ND		ND		ND	
Lyr-R173K	ND		ND		ND	
Lyr-N174L	ND		ND		ND	
Lyr-T391Y	1498±66	53%	527±41	19%	ND	
Lyr-T391Y/S394Y	170±9	6%	26±6	0.9%	ND	
Lyr-A165K/N174L/T391Y	ND		ND		ND	
Wild-type Bar	2397±97	100%	1957±70	82%	192±15	8%
Bar-R174A	ND		ND		ND	
Bar-R174K	ND		ND		ND	
Bar-N175L	ND		ND		ND	
Bar-A393Y	1316±26	55%	ND		44±2	2%
Bar-A166K/N175L/A393Y	ND		ND		ND	

a The conversion yield of D-Lys from L-Lys was defined as 100% for wild-type Lyr and Bar.

ND: Not detectable.

A replacement of the neighboring asparagine with a leucine (N174L in Lyr and N175L in Bar) abolished racemization activity, which supports our hypothesis that this asparagine helps orient the PLP pyridine ring, revealing a comparable role as KCX found in Alr in conjunction with Arg^173^ in Lyr and Arg^174^ in BAR.

Mutation of the inner-layer Thr^391^ of Lyr to tyrosine, which is positioned similarly to the conserved tyrosines in Alrs (Tyr^354^ in BsAlr), reduced the activity of Lyr towards L-Lys to 53% (Table 3). The L-Lys *K_m_* value for this mutant was greater than that for Lyr, whereas the *k_cat_* value for the mutant with L-Lys as the substrate was 42% of Lyr (Table 4). Consequently, racemase activity for the mutant was significantly reduced (∼50% residual activity, Table 3). A larger *K_m_* value towards L-Arg was also measured (Table 4), suggesting that the T391Y mutation might reduce the binding affinity for substrates with bulky side chains. In contrast, the *k_cat_* values were comparable with L-Arg as the substrate, indicating that the transaldimination rate of PLP and L-Arg was unaffected by the mutation. No detectable activity was found when L-Ala was the substrate, implying that alanine still could not access the Lyr-T391Y active site.

**Table 4:** Kinetic Parameters for Wild-type Lyr and Bar, and Their Mutants.

		l-Lys			l-Arg			Specificity constants
		*k_cat_* (min^–1^)	*K_m_* (mM)	*k_cat_/K_m_* (*A*)	*k_cat_* (min^–1^)	*K_m_* (mM)	*k_cat_/K_m_* (*B*)	(*A*)/(*B*)
Wild-type Lyr		3326±68	21.8±0.5	152.6	650±35	14.9±1.0	43.6	3.5
Lyr-T391Y		1384±148	37.6±5.3	36.8	610±12	47.7±1.7	12.8	2.9
Lyr-T391Y/ S394Y		154±11	34.3±4.1	4.5	23±3	75.5±6.2	0.3	15.0
Wild-type Bar		3545±377	25.4±0.6	139.6	2228±14	14.5±1.2	153.7	0.9
Bar-A393Y		1456±131	33.9±3.5	42.9	–	–	–	–

The Bar A393Y mutant exhibited detectable, although reduced activity towards L-Lys (55% residual activity, Table 3). The *k_cat_* value for the mutant with L-Lys as the substrate was reduced (44% residual activity, Table 4), and the *K_m_* value increased 1.6-fold resulting in an overall reduction in activity. A393Y also was less activity toward L-Ala (25% residual activity) as compared with the wild-type enzyme (Table 3) and was inactive toward L-Arg. The tyrosine replacement at residue 393 in Bar therefore significantly reduced racemization activity, particularly when L-Arg was the substrate. Interestingly, *Enterococcus gallinarum* VanT, the C-terminal domain of which shows 31% sequence identity with BsAlr, is a serine racemase that has an asparagine at the site equivalent to position 393 in Bar [32]. Together, these results support the possibility that the residue at this site is critical in defining substrate specificity.

**Figure 6:**
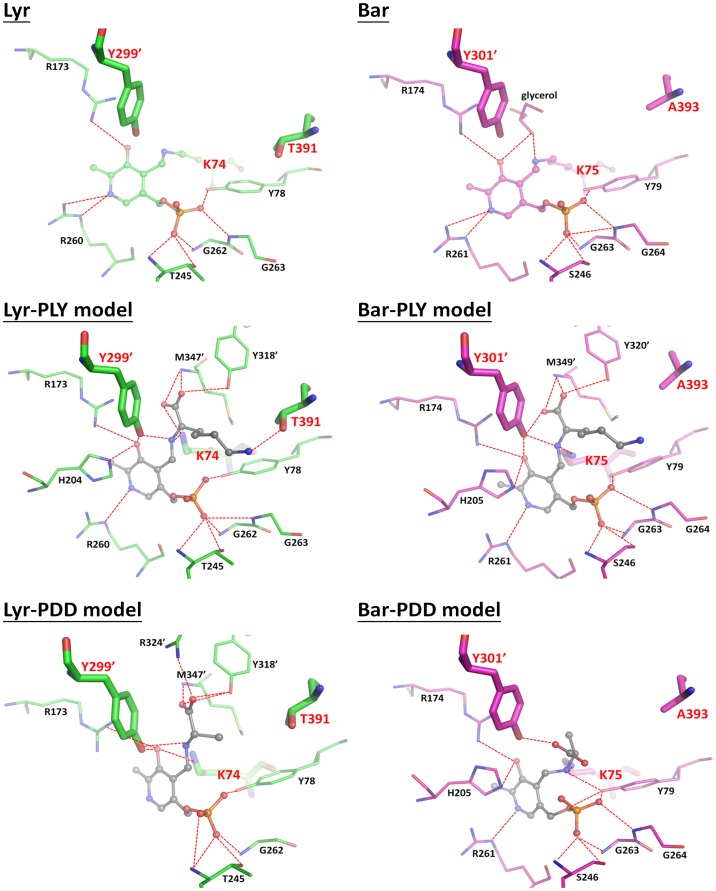
Protein-ligand interactions in crystal structures and docked models (See also Figure S4 and Table S2, S3, S4, S5). Residues of Lyr and Bar are colored green and magenta, respectively. The docked substrates presented as the external aldimine form inside Lyr and Bar are colored gray. The hydrogen bonding is labeled by red dashed lines. Oxygen, nitrogen, and phosphate atoms are colored red, blue, and orange, respectively.

**Figure 7:**
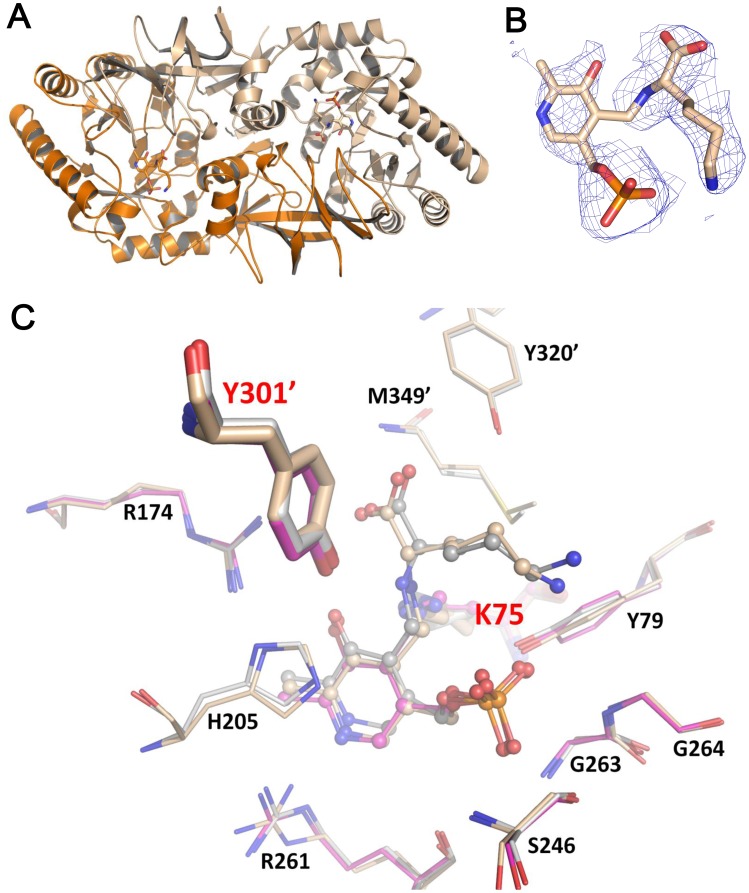
Bar-PLP-L-lysine complex structure. (A) The dimeric Bar is presented as cartoon presentation (chain A, wheat and chain B, orange). PLP and L-lysine are drawn as stick models. (B) The omit *Fo-Fc* electron density map shows PLP, substrate (L-lysine), and the external aldimine linkage, contoured at the 1.0-σ level. (C) Structural comparison of the binding pockets for apo Bar (magenta), L-lysine-liganded Bar (wheat) structures and Bar-PLY (gray) docking model. PLP, substrate L-Lys and catalytic K75 are shown as ball-and-stick models, whereas catalytic Y301′ from the other protomer is drawn as heavy sticks. The rest of surrounding residues are shown as thin sticks. Oxygen, nitrogen, and phosphate atoms are colored in red, blue, and orange, respectively.

We also generated the Lyr triple mutant A165K/N174L/T391Y (Bar, A166K/N175L/A393Y) to mimic the corresponding residues in Alrs. However, no activity was detected towards L-Ala, L-Lys, or L-Arg. Superpositioning of Lyr and the Alrs revealed a subtle difference in the active-site frameworks (Figure 5, see also Figure S1), which might explain the inability of the triple mutant to racemize L-Ala.

Of note, the nearby Bar residue Tyr^396^ in the same helix (α10), for which the phenolic ring also points toward the cavity, has been identified as a valuable engineering site by Kino and colleagues [27]. Substitution of Bar Tyr^396^ with a cysteine or histidine greatly increased racemization activity toward tryptophan [27]. When the corresponding Lyr site was mutated (S394→Y), the activity towards L-Lys was reduced to 31% that of Lyr, whereas activity towards L-Arg increased 2.1-fold [26]. Thus, this site may be also important for catalytic activity in Bar and Lyr. We generated the Lyr double mutant T391Y/S394Y, which exhibited significantly reduced specific activity towards L-Lys (6% residual activity) and toward L-Arg (0.9% residual activity) (Table 3). The *k_cat_* value when L-Lys was the substrate decreased by 22-fold, whereas the *K_m_* value decreased by only 1.6-fold (Table 4). Given these results, it is likely that the Thr^391^ and Ser^394^ side chains, which point toward the entryway, might help accommodate or orient the substrate to effect transaldimination with PLP. An even greater change in *k_cat_* (28-fold) and in *K_m_* (5-fold) was found when L-Arg was the substrate, suggesting that Thr^391^ and Ser^394^ in Lyr (Ala^393^ and Tyr^396^ in Bar) contribute to the binding and orientation of the substrate and therefore to catalytic activity. By considering specificity constants ((*k_cat_*/*K_m_*)Lys/(*k_cat_*/*K_m_*)Arg) in comparing mutations of Lyr and Bar, T391Y has no significant change in substrate specificity (∼2.9 fold) but T391Y/S394Y does enhance the Lys specificity over Arg (∼15.0 fold). This implies that Ser^394^ may be more important than Thr^391^ for defining the substrate specificity in Lyr.

### Docked Lyr and Bar Models

We used an *in silico* docking method to generate the Lyr and Bar aldimine complexes: Lyr-PLP-D-Lys (Lyr-PLY), Lyr-PLP-D-Ala (Lyr-PDD), Bar-PLP-D-Lys (Bar-PLY), and Bar-PLP-D-Ala (Bar-PDD). The top docked poses with minimum RMSDs are shown in Table S2−S5. In Lyr-PLY (Figure 6), a few residues closely interact with cross-linked D-Lys: (i) the catalytic residues Lys^74^ and Tyr^299′^ contact the D-Lys amino nitrogen; (ii) Tyr^318′^ and Met^347′^ interact with the D-Lys carboxyl oxygens; and (iii) Thr^391^ interacts with the D-Lys side-chain Nζ. Similar structural features are also found in Bar-PLY, except for a polar interaction between Ala^393^ and D-Lys (Figure 6).

Conversely, Lyr-PDD and Bar-PDD (Figure 6) contain many fewer polar interactions between D-Ala and PLP. In Lyr-PDD, Lys^74^ does not hydrogen bond with the amino nitrogen of D-Ala. Instead, D-Ala is positioned closer to Arg^324′^, which is why L-Ala is not racemized by Lyr (Table 3). In Bar-PDD, D-Ala is closer to the PLP phosphate, which might also reduce its accessibility to the catalytic residue, Tyr^301′^, and therefore decrease racemase activity.

### The Bar-Lys Complex Structure and Proposed Catalytic Mechanism

The complex-Bar structure was established (Figure 7A, B) by co-crystallizing Bar with L-lysine. The final Bar-L-lysine structure had *R* values of 17.5% (*R_free_* = 21.9%). The apo and liganded Bar structures share the same two-domain architecture and are both composed of the AB-type dimer. Superposition of the apo- and complex-form reveals a RMSD of 0.34 Å for the Cα atoms, demonstrating an overall identical conformation. Moreover, structural comparison of complex-Bar structure and Bar-PLY docking model shows that the reactive intermediates (PLP-lysine) are situated at the comparable position and surrounded by identical residues to form resembling hydrogen-bonding networks (Figure 7C), suggesting the feasibility of the docking approach utilized here.

Two different mechanisms have been previously proposed for the reversible racemization of Alr enzymes: (1) the classical two-base mechanism [9] that describes the involvement of the quinonoid intermediate in the proton transfer between the two catalytic bases; and (2) a revised mechanism that proposes the substrate carboxylate group directly involved in the proton transfer based on the observation of a positively charged arginine (Arg^219^) situated by the pyridine nitrogen atom of PLP in structures of Alr-L-ala and Alr-D-ala [13]. Given the intimate contact between Arg^219^ and the pyridine nitrogen atom of PLP, it is not likely that a quinonoid intermediate proposed in the classical two-base mechanism could be formed. Instead, the substrate carboxylate group that is located in proximity to the two catalytic bases (Lys^39^ and Tyr^265′^ ), proposed in the revised mechanism, is most plausible to serve as the critical moiety to mediate the proton transfer between the two catalytic bases.

The determined Bar-Lys complex structure also shows that the positive guanidium group of Arg^261^ (corresponding to Arg^260^ of Lyr) is close to the pyridine nitrogen atom of PLP (Figure 7C), hence may not favor the formation of the quinonoid intermediate proposed in the classical two-base mechanism. Moreover, the carboxylate group of L-lysine is situated at a position, allowing hydrogen-bonding interactions with both catalytic bases, respectively. Our docking models (Figure 6) are also in good agreement with the liganded Bar-Lys structure. These results together suggest the importance of the substrate carboxylate involved in the catalytic racemization for Lyr and Bar (FigureS3).

In conclusion, the determined structures of apo and L-Lys-liganded Bar present the internal and external aldimine linkages, respectively, as well as the anchored substrate in Bar. The apo Lyr structure has also been established within the internal aldimine linkage. Based on these structures, site-directed mutagenesis, kinetic, and modeling studies, a coordinated response between residues from α-helix 10 and those from the most peripheral region of the binding pocket (corresponding to Glu^208^ and Asp^209^, and Arg^324′^ and Lys^344′^ of Lyr) is essential for lysine-preferred racemases to ensure the substrate orientation, entry and specific binding, hence PLP-dependent catalysis. Our results also shed light on the evolution of PLP-dependent amino acid racemases that retain a converged funnel-like pocket having an outward layer that help anchor the substrate while a base platform for an efficient catalytic racemization. Structural and biochemical investigations in this study provide the key basis to figure out the enzymatic mechanism of Lyr and Bar, which helps to develop and improve the non-antibiotic selection system for transgenic plants.

## Materials and Methods

### Cloning, Expression, and Purification

*P. mirabilis* BCRC10725 *lyr* was PCR amplified from chromosomal DNA using gene specific primers (Table S1). Polymerase chain reaction (PCR) was performed with *pfu* DNA polymerase using a RoboCycler® GRADIENT 96 (Stratagene, La Jolla, CA, USA). Initial denaturation was performed at 95°C for 6 min followed by 30 cycles of denaturation at 95°C for 1 min, annealing at 55°C for 45 s, and primer extension at 72°C for 90 s. The amplified product was inserted into pET21a (Novagen, Inc., USA) to generate pET21a-*lyr*. *Escherichia coli* BL21(DE3) cells were transformed with pET21a-*lyr*, and expression of *lyr* was induced by addition of 0.5 mM isopropyl-β-d-thiogalactopyranoside (IPTG) (final concentration). An overnight culture of *E. coli* BL21(DE3) carrying pET-*lyr* or mutants was inoculated into 500 ml of LB medium containing ampicillin (100 µg/ml) and incubated at 37°C until an OD_600_ of 0.7 was reached. Expression of the gene was induced by the addition of 0.5 mM IPTG and incubated at 17°C for 16 h. The cells were harvested by centrifugation at 10,000×g for 10 min and resuspended in 50 ml of 100 mM potassium phosphate buffer (pH 8.0) containing 150 mM NaCl and disrupted by sonication. The cell debris was pelleted at 10,000×g at 4°C for 10 min and the resulting supernatant was loaded onto a precharged nickel affinity column (HisBind Quick Column, Novagen, Madison, WI, USA). The His_6_-tagged proteins were eluted from the column using a buffer containing 150 mM NaCl, 100 mM imidazole and 100 mM potassium phosphate buffer (pH 8.0). The purified Lyr was verified by SDS-PAGE analysis. Protein concentration was assayed by the Bradford method using bovine serum albumin as the standard [34].

*Pseudomonas putida* DSM84 *bar* was PCR amplified from chromosomal DNA using gene specific primers (Table S1) and inserted into pET21a to generate pET21a-*bar*. The expression and purification of Bar were as described above.

### Site-Directed Mutagenesis

pET21a-*lyr* and pET21a-*bar* served as the templates for overlap extension PCR amplification [35] of mutated genes using the primers listed in Table S1. Each amplified mutated gene was cloned into an *Xba*I-*Xho*I site in pET21a. The recombinant plasmids were individually introduced into *E. coli* NovaBlue or *E. coli* BL21(DE3). After isolating each plasmid, the presence of the desired mutation was confirmed by DNA sequencing.

### Circular Dichroism Analysis

All circular dichroism (CD) measurements described here were made by using 1.0 mm cells. Spectra were recorded at 25°C on AVIV 62A DS spectropolarimeter (Aviv Associates, Lakewood, NJ). The concentration of proteins used for wavelength scanning of CD measurement was 0.08 mg/ml. All spectra were recorded from 195 to 260 nm. Three spectra were recorded and averaged for each protein.

### Enzyme Assay

Lyr and Bar racemase activities were measured using a 0.5 ml mixture of 0.5–1.0 U of purified enzyme, 100 mM CHES-NaOH (pH 9.0), 100 mM of an l-amino acid enantiomer, and 30 µM PLP. Each mixture was incubated at 50°C for 10 min, and the reactions were terminated by heating at 100°C for 10 min. The chiralities of the amino acids were characterized by high-performance liquid chromatography using a Chirobiotic T column (4.0×150 mm, Advanced Separation Technologies, USA) at a flow rate of 0.5 ml/min. The mobile phase was 50% ethanol/50% NaH_2_PO_4_ (pH 4.5). The detection wavelength was 210 nm. One unit of racemase activity was defined as the amount of enzyme that produced 1 µmole of the amino acid enantiomeric product from the enantiomeric reagent per min.

A steady-state kinetics study of the purified enzymes was performed at 50°C with a reaction mixture that contained 100 mM CHES-NaOH (pH 9.0), 30 µM PLP, and an amino acid enantiomer (concentration, 10–150 mM). The *K*_m_ and *k*_cat_ values were determined by plotting the initial velocity as a function of substrate concentration and fitting the plots to the Michaelis-Menten equation.

### Crystallization

Crystallization was performed by the sitting-drop vapor-diffusion method at 20°C. Equal volumes of a protein sample and the reservoir solution were mixed. Initial crystallization screening was automated using a robot Oryx8 (Douglas Instruments, UK) and the reagents of seven sets of crystallization kits: Crystal Screen I and II kits (Hampton Research), Index kit (Hampton Research), Clear Strategy Screen I and II kits (Molecular Dimension), Wizard kit (Emerald), and JB Screen classic HTS I and II kits (Jena Bioscience).

Crystals of Lyr (2.5 mg/ml) were grown in 0.1 M sodium acetate (pH 4.5), 0.2 M lithium sulfate, 30% (w/v) polyethylene glycol (PEG) 8000. Optimized crystals used for diffraction were grown in 0.1 M sodium acetate (pH 4.6), 0.05 M lithium chloride, 29% (w/v) PEG 8000. The crystal diffracted to 1.74 Å, belonged to space group C222_1_, and had unit cell dimensions of *a* = 62.85, *b* = 85.07, *c* = 151.29 Å. The asymmetric unit contained one molecule.

Crystals of Bar (4.7 mg/ml in 10% (v/v) glycerol) were grown in 0.1 M Tris-HCl (pH 8.5), 2.0 M ammonium sulfate. The crystal diffracted to 2.45 Å, belonged to space group C2, and had unit cell dimensions of *a* = 142.25, *b* = 118.74, *c* = 77.78 Å. The asymmetric unit contained two molecules.

Crystals of selenomethionine-derivative Bar (SeMet-Bar) (8.1 mg/ml in 10% (v/v) glycerol) were initially grown in 0.1 M Tris-HCl (pH 8.5), 0.2 M sodium acetate, 25% (w/v) PEG 3350. The random microseed matrix screening method [36] was used to obtain crystals suitable for diffraction. The volumes of the protein sample, reservoir solution, and microseed solution were 0.5 µl, 0.4 µl, and 0.1 µl, respectively. Optimized crystals were grown in 0.1 M Tris-HCl (pH 8.5), 0.2 M lithium sulfate, 1.26 M ammonium sulfate. The SeMet-Bar crystal belonged to space group C2 and had unit cell dimensions of *a* = 140.89, *b* = 118.94, *c* = 74.17 Å. The asymmetric unit contained two molecules.

Crystals of the complex Bar protein (6.1 mg/ml within 4 mM L-lysine) were obtained from the condition using 0.1 M bicine (pH 9.0), 0.1 M sodium chloride, and 20% (w/v) polyethylene glycol monomethyl ether (PEG-MME) 550. The Bar-complex crystal diffracted to 3.10 Å and belonged to space group C2 with the unit cell dimensions *a* = 142.42, *b* = 118.11, *c* = 74.02 Å. There were also two molecules per asymmetric unit.

### X-ray Data Collection and Processing

Crystals were flash frozen in a stream of liquid nitrogen and then screened and characterized using an RU-300 rotating-anode X-ray generator (Rigaku/MSC Inc., USA) at the Macromolecular X-ray Crystallographic Laboratory of National Tsing Hua University, Taiwan. The Lyr dataset was collected at the SPring-8 BL44XU beamline, Japan, with a Bruker-AXS DIP-6040 detector. Bar and SeMet-Bar datasets were collected at the SPXF beamline BL13B1 at the National Synchrotron Radiation Research Center, Taiwan, with an ADSC Quantum-315r CCD area detector. The multi-wavelength anomalous dispersion (MAD) dataset for a single SeMet-Bar crystal was collected at three wavelengths: peak (0.9791 Å), selenium K-edge inflection (0.9792 Å), and high remote (0.9638 Å). The complex-Bar dataset was collected at the SPring-8 BL12B2 beamline, Japan, with the ADSC Quantum-210r CCD area detector. All datasets were indexed, integrated, and scaled using HKL-2000 [37]. Data collection statistics are shown in Table 1.

### Structure Determination and Refinement

Thirteen selenium atoms were located in the asymmetric crystal unit of SeMet-Bar by the program PHENIX [38]. PHENIX was also used for phasing, density modification, and automated model building. The final model included 93.9% of the residues. The first 25 residues of Bar contained the signal peptide and were removed by auto-cleavage. Crystallographic refinement used the maximum-likelihood target function module in REFMAC5 [39], [40]. The apo- and complex-form Bar structures were constructed by MOLREP [39], [40], [41] and were refined using REFMAC5 coupled with ARP/wARP [42], which automatically added water molecules.

The Lyr structure was constructed by the molecular replacement program in PHENIX with the Bar protomer (50% identity) as the template. Crystallographic refinement used the maximum-likelihood target function as implemented in REFMAC5 and coupled to ARP/wARP.

For the Lyr, Bar, complex-Bar and SeMet-Bar structures, 2*Fo-Fc* and *Fo-Fc* maps were produced and inspected after each automated refinement cycle to manually refine the structures with the aid of COOT [43]. The validities of the Lyr, Bar and complex-Bar structures were assessed by PROCHECK [44].

The atomic coordinates and structure factors have been deposited in the Protein Data Bank, Research Collaboratory for Structural Bioinformatics, Rutgers University, New Brunswick, NJ (http://www.rcsb.org/pdb/) under the accession codes 4DZA (Lyr), 4DYJ (Bar), and 4FS9 (Bar-L-lysine).

### Structural and Sequence Comparisons

The Lyr and Bar structures were compared with all protein structures in the DALI server (http://ekhidna.biocenter.helsinki.fi/dali_server/). The structures of Lyr, Bar, complex-Bar, BsAlr [13], SpAlr [19], *Streptomyces lavendulae* Alr (SlAlr, PDB code: 1VFS; [12], and *E. coli* diaminopimelate decarboxylase (PDB code: 1KO0) (no reference), were superimposed by LSQMAN in O [45] to determine the pair-wise RMSD of the Cα atom positions. Q-score, a common measure to present the three-dimensional similarity, was applied to structural comparisons and ranged from 0, where no similarity exists, to 1 where structures are identical [30], [46], [47]. ESPript was used for the combined sequence and secondary structure alignments and figure preparation [48]. PyMol (http://www.pymol.org) was used to prepare the figures containing structures.

### Structural Modeling

Discovery Studio v3.0 (Accelrys Inc., USA) was used to prepare, energy minimize, and refine the Lyr and Bar structures prior to docking D-Ala and D-Lys into Bar and Lyr, respectively, and for molecular dynamics. The default parameters of ChiRotor were used [49] for optimizing both protein side-chain conformations. Energies of the protein models were further minimized using CHARMm [50].

To prepare PLP-D-alanine (PDD) and PLP-D-Lys (PLY) models, D-Ala and D-Lys coordinates were extracted from the BsAlr (PDB code: 1L6G) [13] and *E. coli* D-lysine-diaminopimelate decarboxylase-ligand complexes (PDB code: 1KO0), respectively. LibDock [51] was used in conjunction with its default settings to generate conformations for ligand-containing Lyr and Bar. Hot spots were then aligned to select docked poses, which were then *in situ* minimized by Discovery Studio 3.0 “Conjugate Gradient” [52] to improve the quality of the conformations. All poses were scored, and the top 100-scoring poses were retained for subsequent analyses. Approximately 28% of the poses were retained from the generated 255 poses for each model (Bar: Bar-PDD, Bar-PLY; Lyr: Lyr-PDD, Lyr-PLY). Two additional scoring functions, Potential of Mean Force [53] and LigScore2 scoring [54], [55], were used to compensate for an unfair penalty imposed on the binding-affinity measurement, which resulted because hydrogen atoms were eliminated during LibDock docking. The consensus score for the docked ligands in each protein-ligand model was calculated. Then, CDOCKER [56] with CHARMm forcefield was used to further refine the docked models.

The phosphate moiety in each cognate ligand (PDD or PLY) was fixed in place because its position was superimposable with those in the Alr crystal structures that were complexed with PLP (PDB codes: 1SFT [9], 1BD0 [10], 1L6G [13], 1VFS [12]) as a consequence of an extensive hydrogen-bonding network. When the RMSD of the phosphate in a docked model deviated by ≤1 Å with that of the reference pose (Table S2, S3, S4, S5), the docked model was selected for further study. According to the established mechanism of BsAlr (PDB code: 1L6G [13]), both catalysts (Lys^39^ and Tyr^265′^) were close to the Cα atom of the alanine substrate within hydrogen bonding distances and responsible for the racemization reaction. Hence, the distances between the atom Cα of docking ligands and reactive atoms of two conserved catalyst (Lys:N and Tyr’:O) of Lyr and Bar will be constrained to the extensive hydrogen bonding distance (<4 Å) (Table S2−S5).

To estimate the practicability of the proposed docking procedure, we attempted to self-dock the co-crystal structures complexed with PLP-D-alanine (PDB code: 1L6G [13]) and PLP-D-lysine (PDB code: 1KO0). Top five docked poses with minimum RMSD ≤1 Å were derived. On average, the RMSD of self-docked poses for PLP-D-alanine and PLP-D-lysine are 0.65 Å and 0.84 Å respectively (Figure S4). This suggests that the proposed docking approach is viable.

## Supporting Information

Figure S1 **Related to** **Figure 5:** **Top-view and side-view of the binding cavities of Lyr, Bar, and Alrs.** The views are those obtained by superpositioning the structures of Lyr (green), Bar (magenta), SpAlr (yellow) and BsAlr (cyan). Crucial residues at the substrate entryway are displayed as stick models. PLP-lysine of Lyr, Bar, and SpAlr as well as PLP-D-alanine plus K39 of BsAlr, respectively, are drawn as ball-and-stick models. Spheres of the conserved tyrosine catalysts are colored as magenta. Spheres of positively charged, negatively charged, polar, and non-polar residues are colored as blue, red, pink, and yellow, respectively. Oxygen, nitrogen, and phosphate atoms are colored red, blue, and orange, respectively.
(TIF)

Figure S2 **CD spectra of recombinant proteins.** (A) The wavelength scanning results of Lyr wild-type and mutant proteins. (B) The wavelength scanning results of Bar wild-type and mutant proteins.
(TIF)

Figure S3 **Scheme of the proposed catalytic mechanism of Lyr and Bar.** The substrate, lysine, is colored in red. Catalytic bases are shown as Lys^75^ and Tyr^301′^ of Bar (corresponding to Lys^74^ and Tyr^299′^ of Lyr).
(TIF)

Figure S4 **Related to** **Figure 6:** **Top five results of the self-docking tests.** (A) Self-docking test of 1KO0, a complex structure within PLP and D-lysine. The top five results of docking poses are shown as stick and colored green. (B) Self-docking test of 1L6G, a complex structure within PLP and D-alanine. The top five results of docking poses are shown as stick and colored cyan. The reference pose is shown as ball-and stick and colored gray. Oxygen, nitrogen, and phosphate atoms of reference and docking poses are colored red, blue, and orange, respectively.
(TIF)

Table S1 **Oligonucleotide primers used in this study.**
(DOC)

Table S2 **Related to** **Figure 6:** **Docking results for PLP-D-alanine ligand and Lyr protein.**
(DOC)

Table S3 **Related to** **Figure 6:** **Docking results for PLP-D-alanine ligand and Bar protein.**
(DOC)

Table S4 **Related to** **Figure 6:** **Docking results for PLP-D-lysine ligand and Lyr protein.**
(DOC)

Table S5 **Related to** **Figure 6:** **Docking results for PLP-D-lysine ligand and Bar protein.**
(DOC)

## References

[1] Caparros M, Pisabarro AG, de Pedro MA (1992) Effect of D-amino acids on structure and synthesis of peptidoglycan in Escherichia coli. J Bacteriol 174: 5549–5559. 1512190 10.1128/jb.174.17.5549-5559.1992 PMC206498

[2] Schleifer KH (1975) Chemical structure of the peptidoglycan, its modifiability and relation to the biological activity. Z Immunitatsforsch Exp Klin Immunol 149: 104–117. 126547

[3] Lilley PE, Stamford NP, Vasudevan SG, Dixon NE (1993) The 92-min region of the Escherichia coli chromosome: location and cloning of the ubiA and alr genes. Gene 129: 9–16. 8335265 10.1016/0378-1119(93)90690-5

[4] Esaki N, Walsh CT (1986) Biosynthetic alanine racemase of Salmonella typhimurium: purification and characterization of the enzyme encoded by the alr gene. Biochemistry 25: 3261–3267. 3524677 10.1021/bi00359a027

[5] Oikawa T, Tauch A, Schaffer S, Fujioka T (2006) Expression of alr gene from Corynebacterium glutamicum ATCC 13032 in Escherichia coli and molecular characterization of the recombinant alanine racemase. J Biotechnol 125: 503–512. 16707184 10.1016/j.jbiotec.2006.04.002

[6] Uo T, Yoshimura T, Tanaka N, Takegawa K, Esaki N (2001) Functional characterization of alanine racemase from Schizosaccharomyces pombe: a eucaryotic counterpart to bacterial alanine racemase. J Bacteriol 183: 2226–2233. 11244061 10.1128/JB.183.7.2226-2233.2001 PMC95128

[7] Ono K, Yanagida K, Oikawa T, Ogawa T, Soda K (2006) Alanine racemase of alfalfa seedlings (Medicago sativa L.): first evidence for the presence of an amino acid racemase in plants. Phytochemistry 67: 856–860. 16616264 10.1016/j.phytochem.2006.02.017

[8] Choi SY, Esaki N, Ashiuchi M, Yoshimura T, Soda K (1994) Bacterial glutamate racemase has high sequence similarity with myoglobins and forms an equimolar inactive complex with hemin. Proc Natl Acad Sci USA 91: 10144–10147. 7937852 10.1073/pnas.91.21.10144 PMC44974

[9] Shaw JP, Petsko GA, Ringe D (1997) Determination of the structure of alanine racemase from Bacillus stearothermophilus at 1.9-A resolution. Biochemistry 36: 1329–1342. 9063881 10.1021/bi961856c

[10] Stamper GF, Morollo AA, Ringe D (1998) Reaction of alanine racemase with 1-aminoethylphosphonic acid forms a stable external aldimine. Biochemistry 37: 10438–10445. 9671513 10.1021/bi980692s

[11] Morollo AA, Petsko GA, Ringe D (1999) Structure of a Michaelis complex analogue: propionate binds in the substrate carboxylate site of alanine racemase. Biochemistry 38: 3293–3301. 10079072 10.1021/bi9822729

[12] Noda M, Matoba Y, Kumagai T, Sugiyama M (2004) Structural evidence that alanine racemase from a D-cycloserine-producing microorganism exhibits resistance to its own product. J Biol Chem 279: 46153–46161. 15302886 10.1074/jbc.M404605200

[13] Watanabe A, Yoshimura T, Mikami B, Hayashi H, Kagamiyama H, et al (2002) Reaction mechanism of alanine racemase from Bacillus stearothermophilus: x-ray crystallographic studies of the enzyme bound with N-(5′-phosphopyridoxyl)alanine. J Biol Chem 277: 19166–19172. 11886871 10.1074/jbc.M201615200

[14] LeMagueres P, Im H, Dvorak A, Strych U, Benedik M, et al (2003) Crystal structure at 1.45 A resolution of alanine racemase from a pathogenic bacterium, Pseudomonas aeruginosa, contains both internal and external aldimine forms. Biochemistry 42: 14752–14761. 14674749 10.1021/bi030165v

[15] Watanabe A, Kurokawa Y, Yoshimura T, Kurihara T, Soda K, et al (1999) Role of lysine 39 of alanine racemase from Bacillus stearothermophilus that binds pyridoxal 5′-phosphate. Chemical rescue studies of Lys39–> Ala mutant. J Biol Chem 274: 4189–4194. 9933615 10.1074/jbc.274.7.4189

[16] Watanabe A, Yoshimura T, Mikami B, Esaki N (1999) Tyrosine 265 of alanine racemase serves as a base abstracting alpha-hydrogen from L-alanine: the counterpart residue to lysine 39 specific to D-alanine. J Biochem 126: 781–786. 10502689 10.1093/oxfordjournals.jbchem.a022517

[17] Sun S, Toney MD (1999) Evidence for a two-base mechanism involving tyrosine-265 from arginine-219 mutants of alanine racemase. Biochemistry 38: 4058–4065. 10194319 10.1021/bi982924t

[18] LeMagueres P, Im H, Ebalunode J, Strych U, Benedik MJ, et al (2005) The 1.9 A crystal structure of alanine racemase from Mycobacterium tuberculosis contains a conserved entryway into the active site. Biochemistry 44: 1471–1481. 15683232 10.1021/bi0486583

[19] Im H, Sharpe ML, Strych U, Davlieva M, Krause KL (2011) The crystal structure of alanine racemase from Streptococcus pneumoniae, a target for structure-based drug design. BMC Microbiol 11: 116. 21612658 10.1186/1471-2180-11-116 PMC3146814

[20] Inagaki K, Tanizawa K, Tanaka H, Soda K (1984) Purification and properties of amino acid racemase from Aeromonas punctata subsp. caviae. Prog Clin Biol Res 144A: 355–363. 6427786

[21] Lim YH, Yokoigawa K, Esaki N, Soda K (1993) A new amino acid racemase with threonine alpha-epimerase activity from Pseudomonas putida: purification and characterization. J Bacteriol 175: 4213–4217. 8320235 10.1128/jb.175.13.4213-4217.1993 PMC204851

[22] Huang HT, Kita DA, Davisson JW (1958) Racemization of Lysine by Proteus Vulgaris. J Am Chem Soc 80: 1006–1007.

[23] Chang YF, Adams E (1974) D-lysine catabolic pathway in Pseudomonas putida: interrelations with L-lysine catabolism. J Bacteriol 117: 753–764. 4359655 10.1128/jb.117.2.753-764.1974 PMC285570

[24] Huang HT, Davisson JW (1958) Distribution of lysine racemase in bacteria. J Bacteriol 76: 495–498. 13598707 10.1128/jb.76.5.495-498.1958 PMC290227

[25] Chen IC, Lin WD, Hsu SK, Thiruvengadam V, Hsu WH (2009) Isolation and characterization of a novel lysine racemase from a soil metagenomic library. Appl Environ Microbiol 75: 5161–5166. 19502445 10.1128/AEM.00074-09 PMC2725492

[26] Kuan YC, Kao CH, Chen CH, Chen CC, Hu HY, et al (2011) Biochemical characterization of a novel lysine racemase from Proteus mirabilis BCRC10725. Process Biochem 46: 1914–1920.

[27] Kino K, Sato M, Yoneyama M, Kirimura K (2007) Synthesis of DL-tryptophan by modified broad specificity amino acid racemase from Pseudomonas putida IFO 12996. Appl Microbiol Biotechnol 73: 1299–1305. 17028872 10.1007/s00253-006-0600-6

[28] Chen IC, Thiruvengadam V, Lin WD, Chang HH, Hsu WH (2010) Lysine racemase: a novel non-antibiotic selectable marker for plant transformation. Plant Mol Biol 72: 153–169. 19834817 10.1007/s11103-009-9558-y

[29] Schneider G, Kack H, Lindqvist Y (2000) The manifold of vitamin B6 dependent enzymes. Structure 8: R1–6. 10673430 10.1016/s0969-2126(00)00085-x

[30] Krissinel E, Henrick K (2004) Secondary-structure matching (SSM), a new tool for fast protein structure alignment in three dimensions. Acta Crystallogr D Biol Crystallogr 60: 2256–2268. 15572779 10.1107/S0907444904026460

[31] Au K, Ren J, Walter TS, Harlos K, Nettleship JE, et al (2008) Structures of an alanine racemase from Bacillus anthracis (BA0252) in the presence and absence of (R)-1-aminoethylphosphonic acid (L-Ala-P). Acta Crystallogr F Struct Biol Cryst Commun 64: 327–333. 10.1107/S1744309108007252 PMC2376406 18453697

[32] Patrick WM, Weisner J, Blackburn JM (2002) Site-directed mutagenesis of Tyr354 in Geobacillus stearothermophilus alanine racemase identifies a role in controlling substrate specificity and a possible role in the evolution of antibiotic resistance. Chembiochem 3: 789–792. 12203980 10.1002/1439-7633(20020802)3:8<789::AID-CBIC789>3.0.CO;2-D

[33] Wu D, Hu T, Zhang L, Chen J, Du J, et al (2008) Residues Asp164 and Glu165 at the substrate entryway function potently in substrate orientation of alanine racemase from E. coli: Enzymatic characterization with crystal structure analysis. Protein Sci 17: 1066–1076. 18434499 10.1110/ps.083495908 PMC2386742

[34] Bradford MM (1976) A rapid and sensitive method for the quantitation of microgram quantities of protein utilizing the principle of protein-dye binding. Anal Biochem 72: 248–254. 942051 10.1016/0003-2697(76)90527-3

[35] Chiu WC, You JY, Liu JS, Hsu SK, Hsu WH, et al (2006) Structure-stability-activity relationship in covalently cross-linked N-carbamoyl D-amino acid amidohydrolase and N-acylamino acid racemase. J Mol Biol 359: 741–753. 16650857 10.1016/j.jmb.2006.03.063

[36] Patrick DSS, Stefan AK, Richard AB, Naomi EC, Peter FMB (2011) Random Microseeding: A Theoretical and Practical Exploration of Seed Stability and Seeding Techniques for Successful Protein Crystallization. Cryst Growth Des web publication.

[37] Otwinowski Z, Minor W (1997) Processing of X-Ray Diffraction Data Collected in Oscillation Mode. Method Enzymol 307–326. 10.1016/S0076-6879(97)76066-X 27754618

[38] Adams PD, Grosse-Kunstleve RW, Hung LW, Ioerger TR, McCoy AJ, et al (2002) PHENIX: building new software for automated crystallographic structure determination. Acta Crystallogr D Biol Crystallogr 58: 1948–1954. 12393927 10.1107/s0907444902016657

[39] Collaborative Computational Project, Number 4 (1994) The CCP4 suite: programs for protein crystallography. Acta Crystallogr D Biol Crystallogr 50: 760–763. 15299374 10.1107/S0907444994003112

[40] Murshudov GN, Vagin AA, Dodson EJ (1997) Refinement of macromolecular structures by the maximum-likelihood method. Acta Crystallogr D Biol Crystallogr 53: 240–255. 15299926 10.1107/S0907444996012255

[41] Vagin A, Teplyakov A (1997) MOLREP: an Automated Program for Molecular Replacement. J Appl Cryst 30: 1022–1025.

[42] Lamzin VS, Wilson KS (1993) Automated refinement of protein models. Acta Crystallogr D Biol Crystallogr 49: 129–147. 15299554 10.1107/S0907444992008886

[43] Emsley P, Lohkamp B, Scott WG, Cowtan K (2010) Features and development of Coot. Acta Crystallogr D Biol Crystallogr 66: 486–501. 20383002 10.1107/S0907444910007493 PMC2852313

[44] Laskowski RA, MacArthur MW, Moss DS, Thornton JM (1993) PROCHECK: a program to check the stereochemical quality of protein structures. J Appl Cryst 26: 283–291.

[45] Jones TA, Zou JY, Cowan SW, Kjeldgaard M (1991) Improved methods for building protein models in electron density maps and the location of errors in these models. Acta Crystallogr A 47: 110–119. 2025413 10.1107/s0108767390010224

[46] Chu CH, Lo WC, Wang HW, Hsu YC, Hwang JK, et al.. (2010) Detection and Alignment of 3D Domain Swapping Proteins Using Angle-Distance Image-Based Secondary Structural Matching Techniques. PLoS One 5. 10.1371/journal.pone.0013361 PMC2955075 20976204

[47] Krissinel E (2007) On the relationship between sequence and structure similarities in proteomics. Bioinformatics 23: 717–723. 17242029 10.1093/bioinformatics/btm006

[48] Raghuraman A, Mosier PD, Desai UR (2006) Finding a needle in a haystack: development of a combinatorial virtual screening approach for identifying high specificity heparin/heparan sulfate sequence(s). J Med Chem 49: 3553–3562. 16759098 10.1021/jm060092o PMC2516555

[49] Spassov VZ, Yan L, Flook PK (2007) The dominant role of side-chain backbone interactions in structural realization of amino acid code. ChiRotor: a side-chain prediction algorithm based on side-chain backbone interactions. Protein Sci 16: 494–506. 17242380 10.1110/ps.062447107 PMC2203320

[50] Brooks BR, Bruccoleri RE, Olafson BD, States DJ, Swaminathan S, et al (1983) Charmm - a Program for Macromolecular Energy, Minimization, and Dynamics Calculations. J Comput Chem 4: 187–217.

[51] Diller DJ, Merz KM Jr (2001) High throughput docking for library design and library prioritization. Proteins 43: 113–124. 11276081 10.1002/1097-0134(20010501)43:2<113::aid-prot1023>3.0.co;2-t

[52] Fletcher R, Reeves CM (1964) Function Minimization by Conjugate Gradients. Comput J 7: 149–&.

[53] Muegge I, Martin YC (1999) A general and fast scoring function for protein-ligand interactions: a simplified potential approach. J Med Chem 42: 791–804. 10072678 10.1021/jm980536j

[54] Mayo SL, Olafson BD, Goddard WA (1990) Dreiding - a Generic Force-Field for Molecular Simulations. J Phys Chem 94: 8897–8909.

[55] Krammer A, Kirchhoff PD, Jiang X, Venkatachalam CM, Waldman M (2005) LigScore: a novel scoring function for predicting binding affinities. J Mol Graph Model 23: 395–407. 15781182 10.1016/j.jmgm.2004.11.007

[56] Wu GS, Robertson DH, Brooks CL, Vieth M (2003) Detailed analysis of grid-based molecular docking: A case study of CDOCKER - A CHARMm-based MD docking algorithm. J Comput Chem 24: 1549–1562. 12925999 10.1002/jcc.10306

