# Supplementary figures and images for: Crystal Structures of Lysine-Preferred Racemases, the Non-Antibiotic Selectable Markers for Transgenic Plants
Source: PLoS One. 2012 Oct 31;7(10):e48301. doi: 10.1371/journal.pone.0048301 (PMC3485190; doi:10.1371/journal.pone.0048301)

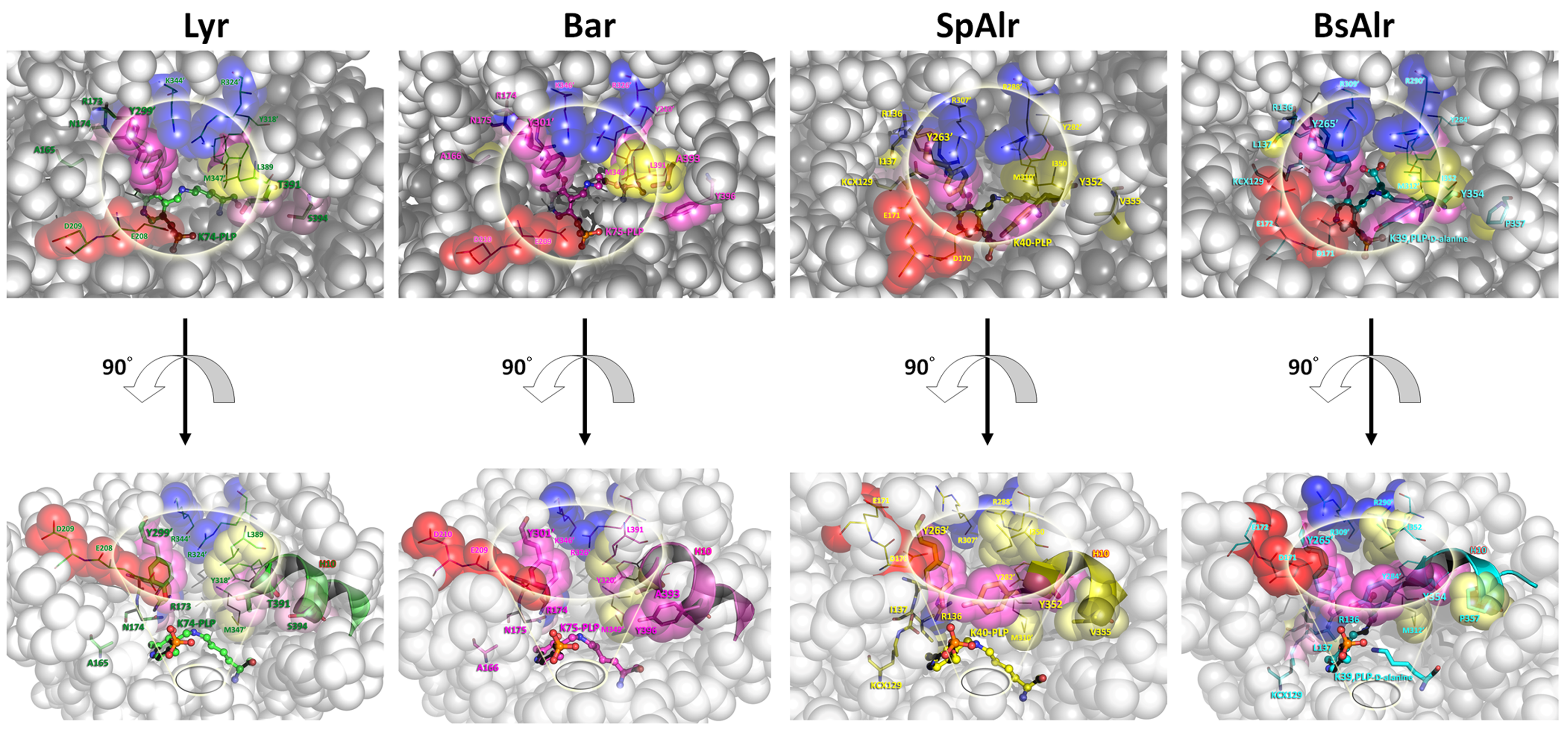

Supplement: Figure S1 — Related to Figure 5: Top-view and side-view of the binding cavities of Lyr, Bar, and Alrs. The views are those obtained by superpositioning the structures of Lyr (green), Bar (magenta), SpAlr (yellow) and BsAlr (cyan). Crucial residues at the substrate entryway are displayed as stick models. PLP-lysine of Lyr, Bar, and SpAlr as well as PLP-D-alanine plus K39 of BsAlr, respectively, are drawn as ball-and-stick models. Spheres of the conserved tyrosine catalysts are colored as magenta. Spheres of positively charged, negatively charged, polar, and non-polar residues are colored as blue, red, pink, and yellow, respectively. Oxygen, nitrogen, and phosphate atoms are colored red, blue, and orange, respectively. (TIF) [file pone.0048301.s001.tif]

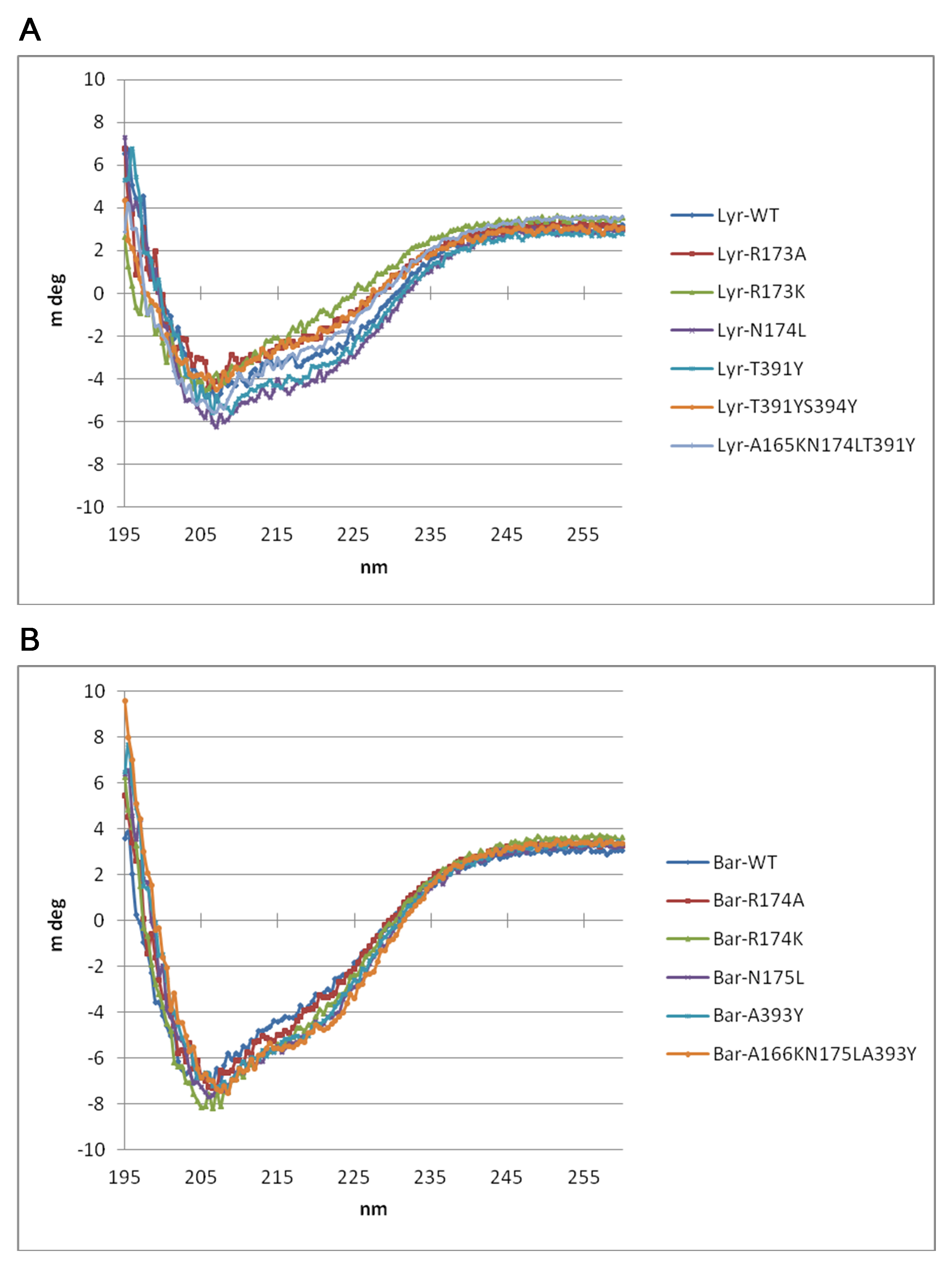

Supplement: Figure S2 — CD spectra of recombinant proteins. (A) The wavelength scanning results of Lyr wild-type and mutant proteins. (B) The wavelength scanning results of Bar wild-type and mutant proteins. (TIF) [file pone.0048301.s002.tif]

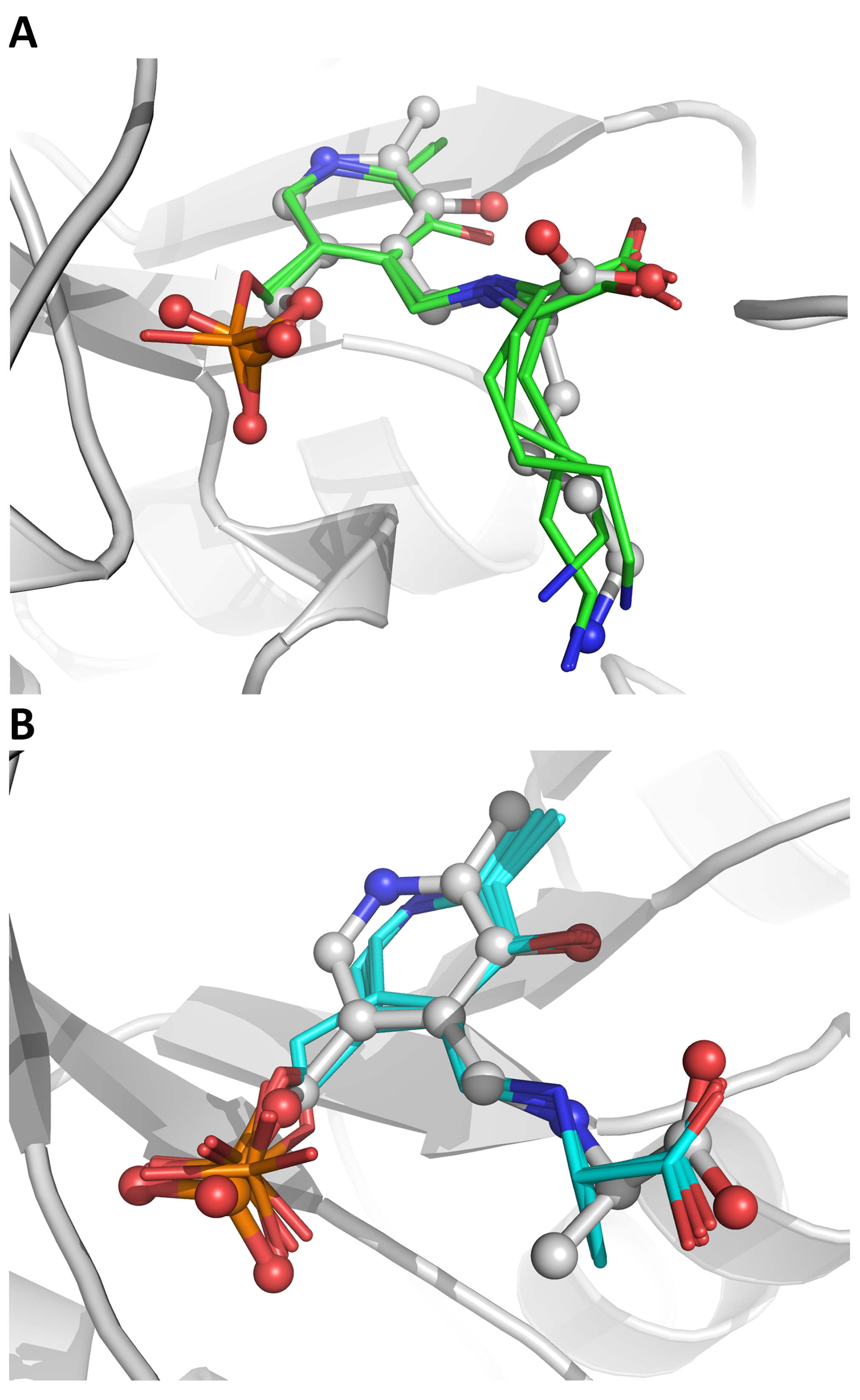

Supplement: Figure S4 — Related to Figure 6: Top five results of the self-docking tests. (A) Self-docking test of 1KO0, a complex structure within PLP and D-lysine. The top five results of docking poses are shown as stick and colored green. (B) Self-docking test of 1L6G, a complex structure within PLP and D-alanine. The top five results of docking poses are shown as stick and colored cyan. The reference pose is shown as ball-and stick and colored gray. Oxygen, nitrogen, and phosphate atoms of reference and docking poses are colored red, blue, and orange, respectively. (TIF) [file pone.0048301.s004.tif]
