# Supplementary material for: Crystal Structures of Lysine-Preferred Racemases, the Non-Antibiotic Selectable Markers for Transgenic Plants
Source: PLoS One. 2012 Oct 31;7(10):e48301. doi: 10.1371/journal.pone.0048301 (PMC3485190; doi:10.1371/journal.pone.0048301)
Supplement: Table S1 — Oligonucleotide primers used in this study. (DOC) [file pone.0048301.s005.doc]

**Table S1. Oligonucleotide primers used in this study.**

| Primer for | Sequence^a^ |
| --- | --- |
| PCR |  |
| *lyr*-F^a^ | 5’-*TCTAGA*AATAATTTTGTTTAACTTTAAGAAGGAGATATACATATGTCTTTAGGTATTCGATATCTTGCGTTA-3’ |
| *lyr*-R^b^ | 5’-CCG*CTCGAG*ATCAACTAATACCCGTTGATTGGTTGCT-3’ |
| *bar*-F^a^ | 5’-*TCTAGA*AATAATTTTGTTTAACTTTAAGAAGGAGATATACATATGCCATTTCGCCGTACACT TCTG-3’ |
| *bar*-R^b^ | 5’-CCG*CTCGAG*GTCGACG AGGATCTTCG GGT-3’ |
| Site-directed mutagenesis | |
| Lyr-R173A-F^c^ | 5’-T TTAGCATTAAATTCAGGTGGCATGTCT GCC AAT GGTTT-3’ |
| Lyr-R173A-R^c^ | 5’- AAACCATTGGCAGACATGCC ACCTGAATTT AATGCTAAA -3’ |
| Lyr-R173K-F^c^ | 5’-T TTA GCA TTAAATTCAGGTGGCATGTCT AAG AAT GGTTT-3’ |
| Lyr-R173K-R^c^ | 5`- AAACCATTCTTAGACATGCC ACCTGAATTT AATGCTAAA -3’ |
| Lyr-N174L-F^c^ | 5’-T TTA GCA TTAAATTCAGGTGGCATGTCTCGTCTG GGTTT-3’ |
| Lyr-N174L-R^c^ | 5’-AAACCCAGAC GAGACATGCC ACCTGAATTT AATGCTAAA-3’ |
| lyr-S391N- R^bc^ | 5’-CGG*CTCGAG*ATCAACT AATACCCGTT GATTGGTTGC TCCCCATAAA ATTGACATTT CATAAAACAA-3’ |
| Lyr-S391N&S394Y-R^bc^ | 5’-CGG*CTCGAG*ATCAACT AATACCCGTTGATTGGTTGC TCCCCATAAA ATA TACATTTCATAAAACAA-3’ |
| Lyr-A165K&N174L-F^c^ | 5`-T TTA AAGTTAAATTCAGGTGGCATGTCTCGTCTG GGTTT-3’ |
| Lyr-A165K&N174L-R^c^ | 5’-AAACCCAGAC GAGACATGCC ACCTGAATTT AACTTTAAA-3’ |
| Bar- R174K-F^c^ | 5’-T TCCAGC GGCATGAGCAAGAACGGCGTGGAA-3’ |
| Bar-R174K-R^c^ | 5’- TTCCACGCCG TTCTTGCTCATGCCGCTGGAA-3’ |
| Bar- R174A-F^c^ | 5’-T TCCAGCGGCATGAGCGCCAACGGCGTGGAA-3’ |
| Bar-R174A-R^c^ | 5’-TTCCACGCCGTTGGCGCTCATGCCGCTGGA A-3’ |
| Bar-N175L- F^c^ | 5’-TTCCAGCGGCATGAGCCGCCTGGGCGTGGAA -3’ |
| Bar-N175L- R^c^ | 5’- TTCCACGCCCAGGCGGCTCATGCCGCTGGA A-3’ |
| Bar-A166K&N175L-F^c^ | 5’-CATGAAGCTCAATTCCAGCGGCATGAGCCGCCTGGGCGTGGA-3’ |
| Bar-A166K&N175L-R^c^ | 5’- TCCACGCCCAGGCGGCTCAT GCCGCTGGAA TGAGCTTCATG -3’ |
| BAR-A393Y-R^bc^ | 5’-CGG*CTCGAG*GTCGACGAGTATCTTCGGGTTGGAACTGCCCCATACGGTG TACAGATCGTAGAGCAG-3’ |
| BAR-A393Y&Y396C-R^bc^ | 5’-CCG*CTCGAG*GTCGACGAGGATCTTCGGGTTGGAACTGCCCCATACGGTGCACAGATCGTAGAGCAG-3-3’ |

^a^*Xba*I site is indicate italics

^b^*Xho*I site is indicate italics

^c^ Mutated nucleotides are underlined
