# Supplementary material for: Crystal Structures of Lysine-Preferred Racemases, the Non-Antibiotic Selectable Markers for Transgenic Plants
Source: PLoS One. 2012 Oct 31;7(10):e48301. doi: 10.1371/journal.pone.0048301 (PMC3485190; doi:10.1371/journal.pone.0048301)
Supplement: Table S2 — Related to Figure 6: Docking results for PLP-D-alanine ligand and Lyr protein. (DOC) [file pone.0048301.s006.doc]

**Table S2,** related to Figure 6. Docking results for PLP-D-alanine ligand and Lyr protein.

| Pose ID^a^ | RMSD_K74:N_^b^ | RMSD_Y299’:O_^b^ | wRMSD^a^ |
| --- | --- | --- | --- |
| 12 | 5.061 | 3.414 | 1.595* |
| 14 | 4.976 | 3.436 | 1.598 |
| 34 | 5.469 | 3.373 | 1.612 |
| 13 | 5.082 | 3.415 | 1.618 |
| 16 | 5.200 | 3.407 | 1.655 |
| 15 | 5.217 | 3.408 | 1.667 |
| 35 | 5.624 | 3.470 | 2.008 |

a. 7 poses of D-alanine combined with PLP (PLP-D-alanine) docked into Lyr binding site were obtained from 82 poses which were generated using CDOCKER. The constraint distance of both phosphate groups among the pose and idea PLP-D-alanine (derived from superimposing Lyr and the liganded Alr structure (PDB code: 1L6G)) is less than 1 Å. The pose with the smallest 1.595 of wRMSD (with the star marker) was selected as the final solution for the subsequent analysis. wRMSD is the abbreviation of RMSD of whole heavy atoms among two compounds with identical atoms name.

b. All distances between C17 atoms of the poses and N (K74) & O (Y299’) atoms of Lyr were calculated. In such case, only the O atom of Y299’ of Lyr could form hydrogen bound (<4 Å) with C17 atoms of the selected poses. RMSDs range from 3.373 to 3.436. The formula of RMSD is$\sqrt{N^{-1}\sum_{i=1}^{i=N} \delta_{i}^{2}}$, where *δ* is the distance between *N* pairs of equivalent atoms.
