# Supplementary material for: Crystal Structures of Lysine-Preferred Racemases, the Non-Antibiotic Selectable Markers for Transgenic Plants
Source: PLoS One. 2012 Oct 31;7(10):e48301. doi: 10.1371/journal.pone.0048301 (PMC3485190; doi:10.1371/journal.pone.0048301)
Supplement: Table S3 — Related to Figure 6: Docking results for PLP-D-alanine ligand and Bar protein. (DOC) [file pone.0048301.s007.doc]

**Table S3,** related to Figure 6. Docking results for PLP-D-alanine ligand and Bar protein.

| Pose ID^a^ | RMSD_K75:N_^b^ | RMSD_Y301’:O_^b^ | wRMSD^a^ |
| --- | --- | --- | --- |
| 25 | 3.918 | 3.391 | 1.325* |
| 46 | 4.112 | 3.750 | 1.460 |
| 41 | 4.816 | 3.484 | 1.676 |
| 40 | 4.825 | 3.478 | 1.696 |
| 44 | 4.890 | 3.490 | 1.742 |
| 2 | 5.163 | 3.498 | 1.864 |
| 24 | 4.748 | 3.494 | 1.884 |
| 42 | 5.094 | 3.517 | 1.887 |
| 21 | 4.736 | 3.477 | 1.914 |
| 22 | 4.736 | 3.539 | 1.916 |
| 23 | 4.756 | 3.530 | 1.925 |
| 43 | 5.116 | 3.528 | 1.934 |
| 3 | 5.271 | 3.521 | 1.952 |
| 5 | 5.227 | 3.564 | 1.963 |
| 20 | 4.841 | 3.504 | 1.965 |
| 4 | 5.375 | 3.532 | 1.999 |
| 7 | 5.383 | 3.654 | 2.083 |
| 50 | 5.365 | 3.548 | 2.098 |
| 57 | 5.448 | 3.626 | 2.143 |
| 51 | 5.389 | 3.620 | 2.154 |
| 56 | 5.469 | 3.619 | 2.155 |
| 55 | 5.351 | 3.565 | 2.283 |
| 11 | 5.576 | 5.876 | 4.850 |

1. 23 poses of D-alanine combined with PLP (PLP-D-alanine) docked into Bar binding site were obtained from 67 poses which were generated using CDOCKER. The constraint distance of both phosphate groups among the pose and idea PLP-D-alanine (derived from superimposing Bar and the liganded Alr structure (PDB code: 1L6G)) is less than 1 Å. The pose with the smallest 1.325 of wRMSD (with the star marker) was selected as the final solution for the subsequent analysis. The formula of RMSD and the definition of wRMSD are described in Table S2.

b. All distances between C17 atoms of the poses and the N (K75) & O (Y301’) atoms of Bar were calculated. In such case, both N (K75) and O (Y301’) atoms form hydrogen bounds (<4 Å) with the C17 atom of the pose 25 (with star marker). The RMSDs are 3.918 and 3.391 respectively.
