# Supplementary material for: Crystal Structures of Lysine-Preferred Racemases, the Non-Antibiotic Selectable Markers for Transgenic Plants
Source: PLoS One. 2012 Oct 31;7(10):e48301. doi: 10.1371/journal.pone.0048301 (PMC3485190; doi:10.1371/journal.pone.0048301)
Supplement: Table S4 — Related to Figure 6: Docking results for PLP-D-lysine ligand and Lyr protein. (DOC) [file pone.0048301.s008.doc]

**Table S4,** related to Figure 6. Docking results for PLP-D-lysine ligand and Lyr protein.

| Pose ID^a^ | RMSD_K74:N_^b^ | RMSD_Y299’:O_^b^ | wRMSD^a^ |
| --- | --- | --- | --- |
| 29 | 3.581 | 3.202 | 1.967* |
| 2 | 3.478 | 3.142 | 2.309 |
| 1 | 3.541 | 3.136 | 2.447 |
| 52 | 5.751 | 3.100 | 2.454 |
| 53 | 5.741 | 3.086 | 2.507 |
| 54 | 5.620 | 3.142 | 2.510 |
| 74 | 4.937 | 3.675 | 2.709 |
| 71 | 4.706 | 3.679 | 2.710 |
| 75 | 4.501 | 4.175 | 2.712 |
| 73 | 4.838 | 3.600 | 2.771 |
| 72 | 4.682 | 3.714 | 2.916 |

a. 11 poses of D-lysine combined with PLP (PLP-D-lysine) docked into Lyr binding site were obtained from 86 poses which were generated using CDOCKER. The constraint distance of both phosphate groups among the pose and idea PLP-D-lysine (derived from superimposing Lyr and a liganded diaminopimelate decarboxylase protein (PDB code: 1KO0)) is less than 1 Å. The pose with the smallest 1.967 of wRMSD (with the star marker) was selected as the final solution for the subsequent analysis. The formula of RMSD and the definition of wRMSD are described in Table S2.

b. All distances between C17 atoms of the poses and the N (K74) and O (Y299’) atoms of Lyr were calculated. In such case, both N (K74) and O (Y299’) atoms form hydrogen bounds (<4 Å) with C17 atoms of the poses 29, 2, and 1.
